# Protection of cell therapeutics from antibody-mediated killing by CD64 overexpression

**DOI:** 10.1038/s41587-022-01540-7

**Published:** 2023-01-02

**Authors:** Alessia Gravina, Grigol Tediashvili, Raja Rajalingam, Zoe Quandt, Chad Deisenroth, Sonja Schrepfer, Tobias Deuse

**Affiliations:** 1 Transplant and Stem Cell Immunobiology (TSI) Laboratory, Department of Surgery, University of California, San Francisco, San Francisco, CA USA; 2 Immunogenetics and Transplantation Laboratory, Department of Surgery, University of California, San Francisco, San Francisco, CA USA; 3 Department of Medicine, Division of Diabetes, Endocrinology and Metabolism, University of California, San Francisco, San Francisco, CA USA; 4 United States Environmental Protection Agency, Center for Computational Toxicology & Exposure, Durham, NC USA

**Keywords:** Stem-cell research, Immune evasion

## Abstract

Allogeneic cell therapeutics for cancer therapy or regenerative medicine are susceptible to antibody-mediated killing, which diminishes their efficacy. Here we report a strategy to protect cells from antibody-mediated killing that relies on engineered overexpression of the IgG receptor CD64. We show that human and mouse iPSC-derived endothelial cells (iECs) overexpressing CD64 escape antibody-dependent cellular cytotoxicity (ADCC) and complement-dependent cytotoxicity from IgG antibodies in vitro and in ADCC-enabled mice. When CD64 expression was combined with hypoimmune genetic modifications known to protect against cellular immunity, B2M^−/−^CIITA^−/−^ CD47/CD64-transgenic iECs were resistant to both IgG antibody-mediated and cellular immune killing in vitro and in humanized mice. Mechanistic studies demonstrated that CD64 or its intracellularly truncated analog CD64t effectively capture monomeric IgG and occupy their F_c_, and the IgG bind and occupy their target antigens. In three applications of the approach, human CD64t-engineered thyroid epithelial cells, pancreatic beta cells and CAR T cells withstood clinically relevant levels of graft-directed antibodies and fully evaded antibody-mediated killing.

## Abstract (web summary)

Hypoimmune engineering of cell therapies is extended to protection from host antibodies.

## Main

The concept of antibody-mediated rejection (AMR) after solid organ transplantation became a focus in transplant research in the 1990s, decades after the concept of cellular rejection had been widely accepted. A hallmark of AMR is the presence of graft-specific antibodies^1^ in combination with graft damage. The emergence of such antibodies occurs despite the use of guideline-driven systemic immunosuppression. Outside of transplantation, some autoimmune diseases are characterized by autoantibodies that mediate the destruction of the target cells and persist even after the affected cell population has vanished. The emergence of antibodies against allogeneic cell therapeutics has been observed in clinical trials^2–4^. Cancer therapy with chimeric antigen receptor (CAR) T cells induces antibodies, especially if tumor cell types other than B cells or plasma cells are targeted^5^. It is, therefore, likely that most allogeneic cellular grafts for long-term regenerative or oncology indications in immunocompetent patients will eventually experience some form of antibody-mediated killing. We, therefore, sought to develop a gene engineering approach that provides antibody protection for cell therapeutics.

For both antibody-mediated cellular cytotoxicity (ADCC) and complement-dependent cytotoxicity (CDC), antibodies of the IgG class mediate target cell killing by binding an epitope via their antigen-binding fragments (F_ab_) and activating effector cells or complement via their free fragment crystallizable domain (F_c_). We hypothesized that forced overexpression of the high-affinity receptor for IgG F_c_ (CD64) on graft cells would capture monomeric IgG F_c_ and make F_c_ inaccessible for effector cells or complement. IgG against epitopes expressed on these cells could bind and occupy those. We found that the protection that CD64 overexpression reliably established was effective against ADCC and CDC, was agnostic to the specific type of cell and was applicable to three clinically relevant cell therapeutics.

## Results

### Cd64-expressing mouse iECs are protected from antibody-mediated killing

Mouse C57BL/6 (B6) induced pluripotent stem cells (iPSCs) were differentiated into B6 iECs, and the cells were transduced with lentiviral particles to express the mouse Cd64 transgene. B6 iECs^Cd64^ were able to bind free mouse IgG2a F_c_ in a concentration-dependent manner (Supplementary Fig. 1a,b). In mice, IgG2a and IgG2b are the main antibody isotypes mediating ADCC and CDC. For these F_c_ binding assays, antibodies are used that are specific for an epitope that is not expressed on the cells to avoid any specific F_ab_ binding. The flow cytometry signal then only measures antibodies captured via F_c_. For in vitro killing assays, B6 iECs and B6 iECs^Cd64^ were grown on electrode plates for real-time impedance cytotoxicity assays with B6 natural killer (NK) cells as effector cells (ADCC) or B6 serum (CDC). In this highly sensitive assay, target cell death leads to a disruption of the cell covering of electrodes with a decrease of impedance and drop of the plotted cell index curve.

We used a mouse IgG2a antibody against the B6 major histocompatibility complex (MHC) haplotype H-2^b^ and found that it effectively mediates ADCC and CDC against B6 iECs. Engineered B6 iECs^Cd64^ were fully protected against ADCC and CDC (Supplementary Fig. 1c,d).

In a next step, we modified B6 *B2m*^*−/−*^*Ciita*^*−/−*^Cd47^+^ hypoimmune (HIP; Supplementary Fig. 2a) iECs^6^ to additionally express human CD64 (B6 HIP iECs^CD64^). HIP cells are protected from allogeneic innate and adaptive immune cell killing^6,7^ but are potentially susceptible to antibody-mediated killing. To design a very stringent model, target cells were additionally transduced to express human CD52, the target for the highly cytotoxic anti-CD52 antibody alemtuzumab (Supplementary Fig. 2b). B6 HIP iECs^CD64^ showed human IgG1 F_c_ capture ability in a concentration-dependent manner (Supplementary Fig. 2c). In ADCC and CDC assays with anti-CD52, B6 HIP iECs^CD52^ were killed by mouse NK cells and complement even at low antibody concentrations. This confirmed the high cytotoxic capacity of alemtuzumab and its functional compatibility with mouse NK cells and complement. B6 HIP iECs^CD52,CD64^ were fully resistant against ADCC and CDC across the anti-CD52 concentration spectrum (Supplementary Fig. 2d,e).

Next, grafts of 1 million firefly luciferase-positive (Luc^+^) B6 HIP iECs^CD52^ and B6 HIP iECs^CD52,CD64^ were transplanted subcutaneously into Rag-1-deficient mice. This strain lacks mature B and T cells but has functional NK cells^8^ and serves well as an ADCC in vivo model. Two alemtuzumab doses at 1 mg each were injected into the peritoneum on post-transplant days 0 and 3. Graft survival was followed by bioluminescence imaging (BLI) (Supplementary Fig. 2f). We saw the B6 HIP iECs^CD52^ grafts vanish within approximately 1 week, whereas B6 HIP iECs^CD52,CD64^ grafts survived and were protected from ADCC (Supplementary Fig. 2g,h). The ability of CD64 to capture F_c_ was thus shown to add antibody protection to mouse HIP cells in vivo and in vitro.

### CD64-expressing human iECs are protected from antibody-mediated killing

**Fig. 1:**
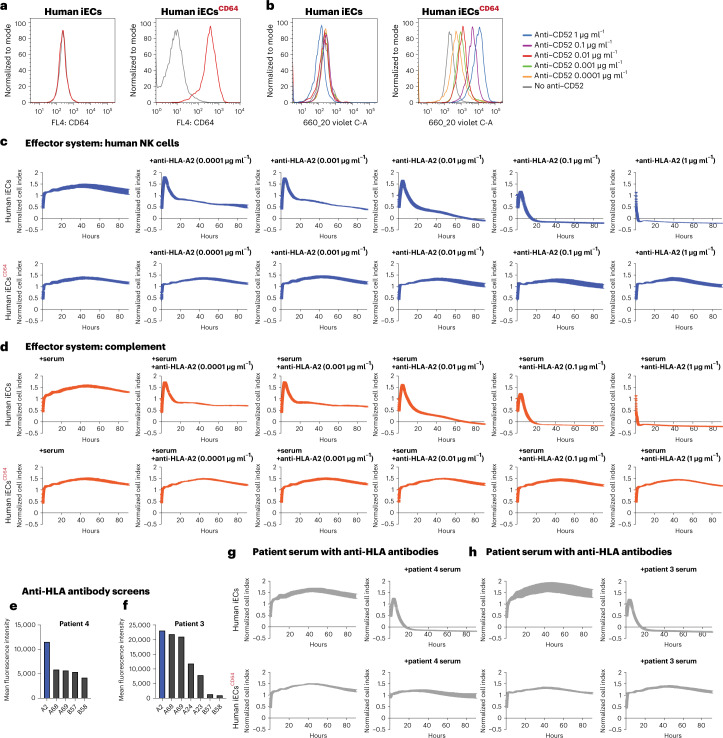
Capturing of IgG F_c_ protects iECs from HLA antibody-mediated killing. **a**, Flow cytometry histograms for CD64 expression on human iECs and iECs^CD64^ (representative graph of two independent experiments). **b**, Flow cytometry histograms for the binding of free IgG1 F_c_ (anti-CD52, alemtuzumab; representative graph of two independent experiments). **c**,**d**, Human iECs and iECs^CD64^ were challenged in impedance NK cell ADCC (**c**) and CDC (**d**) assays with different concentrations of an anti-HLA-A2 IgG1 antibody (mean ± s.d.; three independent replicates per group and timepoint). **e**,**f**, Single antigen bead assay results for HLA class I and II antibodies in transplant recipients 4 (**e**) and 3 (**f**) experiencing AMR. **g**,**h**, Human iECs and iECs^CD64^ were incubated with serum from patient 4 (**g**) and patient 3 (**h**) in impedance CDC assays (mean ± s.d.; three independent replicates per group and timepoint).

Human iECs of the HLA-A*02:01 genotype were transduced to overexpress human CD64 using lentiviral particles, and cells with high CD64 expression (iECs^CD64^) were enriched using cell sorting (Fig. 1a). Their ability to capture human IgG1 F_c_ in a concentration-dependent manner was shown (Fig. 1b). In vitro killing assays were performed using a recombinant human anti-HLA-A2 IgG1 antibody^9^ with human NK cells as effector cells (ADCC) or human serum (CDC). Whereas iECs underwent increasingly rapid killing via NK cell ADCC (Fig. 1c) and CDC (Fig. 1d) with increasing anti-HLA-A2 concentrations, iECs^CD64^ were fully protected, and no target cell killing was observed. The anti-HLA-A2 antibody in CDC assays was added to complete human serum containing 6–16 mg ml^−1^ of total IgG, so that it had to compete with all serum IgG for target cell CD64. To assess whether this antibody protection would hold up in patients experiencing clinically relevant AMR, we identified two transplant recipients with anti-HLA-A2 mean fluorescence intensities (MFIs) >10,000 in single-antigen bead assays (Fig. 1e,f) and clinical signs of AMR of their grafts. Patient serum samples were incubated with iECs and iECs^CD64^ (Fig. 1g,h). Whereas iECs were very rapidly killed in this CDC assay, iECs^CD64^ again were completely protected without any signs of cell damage, as shown by the steady impedance for 90 hours. To next evaluate non-HLA antibody killing, we used a recombinant human IgG1 antibody against MHC class I-related sequence A (MICA)^10,11^, which is constitutively expressed on ECs^12–14^ (Supplementary Fig. 3a,b). We again observed that iECs were very susceptible to NK cell ADCC and CDC, whereas iECs^CD64^ were fully protected. These experiments show that high CD64 expression can protect target cells from HLA and non-HLA antibody-mediated killing.

### Generation of completely immune-evasive human cells

**Fig. 2:**
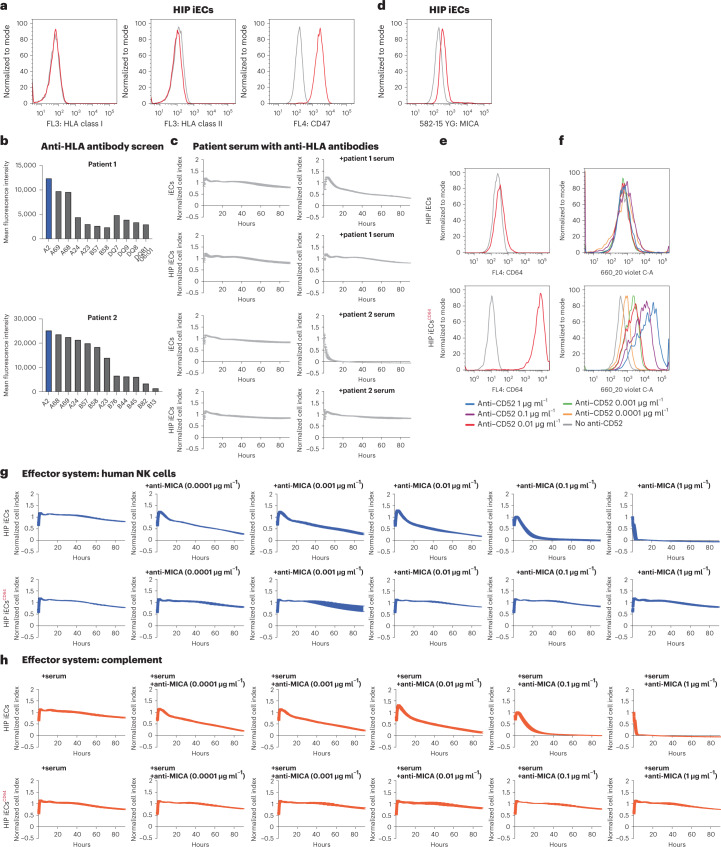
Completely immune-evasive human HIP iECs^CD64^. **a**, The HIP immune phenotype of HLA class I and II deficiency and CD47 overexpression was confirmed in flow cytometry (representative graph of two independent experiments). **b**, Single antigen bead assay results for HLA class I and II antibodies in transplant recipients 1 and 2 experiencing AMR. **c**, Human iECs and HIP iECs were incubated with serum from both patients in impedance CDC assays (mean ± s.d.; three independent replicates per group and timepoint). **d**, Flow cytometry histogram for MICA expression on human HIP iECs (representative graph of two independent experiments). **e**, Flow cytometry histograms for CD64 expression on human HIP iECs and HIP iECs^CD64^ (representative graph of two independent experiments). **f**, Flow cytometry histograms for the binding of free IgG1 F_c_ (anti-CD52, alemtuzumab; representative graph of two independent experiments). **g**,**h**, Human HIP iECs and HIP iECs^CD64^ were challenged in impedance NK cell ADCC (**g**) and CDC (**h**) assays with different concentrations of an anti-MICA IgG1 antibody (mean ± s.d.; three independent replicates per group and timepoint).

Human *B2M*^*−/−*^*CIITA*^*−/−*^CD47^+^ (HIP) iECs^6^ (Fig. 2a) are naturally protected from HLA antibodies given their lack of HLA class I and II expression. To confirm that, human iECs and human HIP iECs (both HLA-A2*02:01) were incubated with serum from two more transplant recipients with high anti-HLA-A2 MFIs and clinical signs of AMR (Fig. 2b). We saw CDC killing of iECs but not of HIP iECs. The killing was faster in the serum with higher anti-HLA-A2 MFI levels (Fig. 2c). The non-HLA epitope MICA is constitutively expressed on human HIP iECs (Fig. 2d), which leaves them susceptible to anti-MICA antibodies. We then generated CD64-expressing HIP iECs (HIP iECs^CD64^), which were able to capture human IgG1 F_c_ in vitro (Fig. 2e,f). As expected, HIP iECs underwent increasingly rapid NK cell ADCC and CDC (Fig. 2g,h) with increasing concentrations of the humanized IgG1 anti-MICA antibody. Human HIP iECs^CD64^ were completely resistant against anti-MICA ADCC and CDC. Because both HIP iECs and HIP iECs^CD64^ were also positive for the rhesus D blood type antigen, ADCC and CDC assays were performed with a humanized IgG1 anti-Rh(D) antibody (Supplementary Fig. 4a,b). Whereas HIP iECs were killed in both assays, HIP iECs^CD64^ were resistant against anti-Rh(D) cytotoxicity. We next tested the binding affinities of IgG isotypes and found that CD64 shows high affinity to monomeric IgG1, IgG3 and IgG4 (Supplementary Fig. 3c,d). Although IgG1 is the most relevant antibody isotype in solid organ AMR, other isotypes have been documented^15^. Kidney transplant patients with donor-specific antibodies experiencing AMR were shown to predominantly have complement-fixing IgG1 and IgG3 for HLA and non-HLA antibodies^16–18^. In a clinical setting, CD64 overexpression should thus protect from the vast majority of human IgG.

**Fig. 3:**
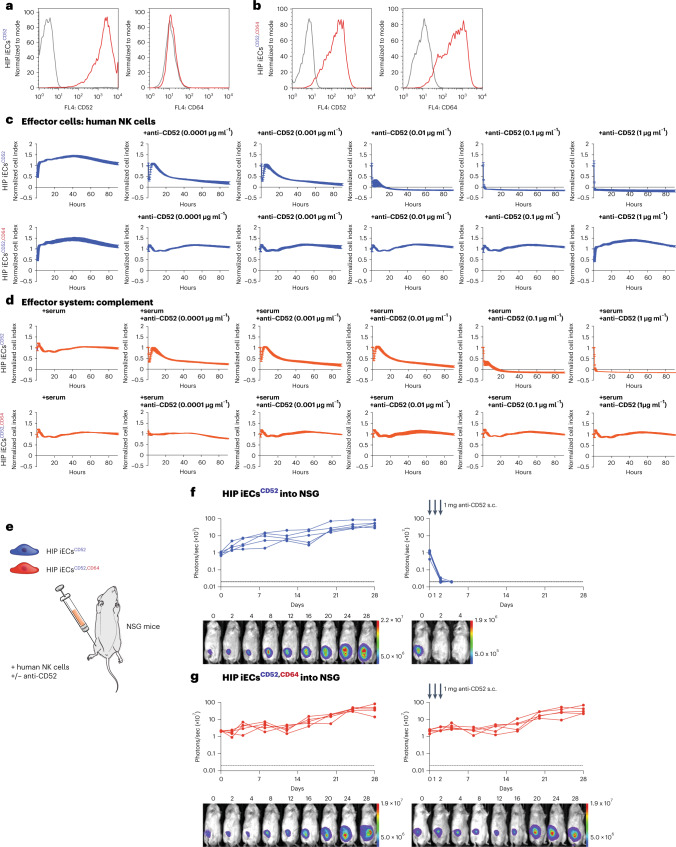
CD64 expression protects human HIP iECs^CD52,CD64^ from non-HLA antibody killing in vitro and in vivo. **a**, Flow cytometry histograms for CD52 and CD64 expression on human HIP iECs^CD52^ (representative graphs of two independent experiments). **b**, Flow cytometry histograms for CD52 and CD64 expression on human HIP iECs^CD52,CD64^ (representative graphs of two independent experiments). **c**,**d**, Human HIP iECs^CD52^ and HIP iECs^CD52,CD64^ were challenged in impedance NK cell ADCC (**c**) and CDC (**d**) assays with different concentrations of an anti-CD52 IgG1 antibody (mean ± s.d.; three independent replicates per group and timepoint). **e**, 5 × 10^4^ HIP iECs^CD52^ or HIP iECs^CD52,CD64^ were injected subcutaneously into NSG mice with 10^6^ human NK cells. Some groups received three subcutaneous doses of alemtuzumab 1 mg on days 0, 1 and 2. **f**,**g**, BLI signals of HIP iECs^CD52^ (**f**) and HIP iECs^CD52,CD64^ (**g**) were followed (all individual mice were plotted, and BLI pictures of one representative mouse per group are shown). s.c., subcutaneous.

We next aimed to verify antibody evasiveness with the highly cytotoxic human anti-CD52 IgG1 alemtuzumab and generated HIP iECs^CD52^ and HIP iECs^CD52,CD64^ that express its CD52 target (Fig. 3a,b). In vitro ADCC and CDC assays with alemtuzumab and allogeneic human NK cells confirmed the high cytotoxicity of alemtuzumab, already killing human HIP iECs^CD52^ at 0.0001 μg ml^−1^ (Fig. 3c,d). Human HIP iECs^CD52,CD64^ on the other side were resistant against ADCC and CDC, thus withstanding 100,000-times higher alemtuzumab concentrations. In an NSG mouse in vivo killing assay, 5 million HIP iECs^CD52^ and HIP iECs^CD52,CD64^ with different fluorescence labels were simultaneously injected intraperitoneally together with 100 million allogeneic human NK cells or macrophages and alemtuzumab (Supplementary Fig. 5a). After 48 hours, the peritoneal cells were recovered, and we saw that, with increasing alemtuzumab concentrations, we almost exclusively recovered HIP iECs^CD52,CD64^, whereas HIP iECs^CD52^ had vanished (Supplementary Fig. 5b,c). We next assessed cell survival in an NSG mouse in vivo ADCC killing assay using BLI. A total of 5 × 10^4^ HIP iECs^CD52^ or HIP iECs^CD52,CD64^ were injected subcutaneously with 1 million human NK cells with or without alemtuzumab (Fig. 3e). Both cell populations showed consistent survival when no antibody was added. With anti-CD52 administered at 1-mg doses on days 0, 1 and 2, HIP iECs^CD52^ were expeditiously rejected (Fig. 3f). The survival of HIP iECs^CD52,CD64^, in contrast, remained completely unaffected (Fig. 3g). Together, these data show that CD64 expression is highly effective to prevent HIP iEC killing in vitro and in vivo by various non-HLA antibodies.

**Fig. 4:**
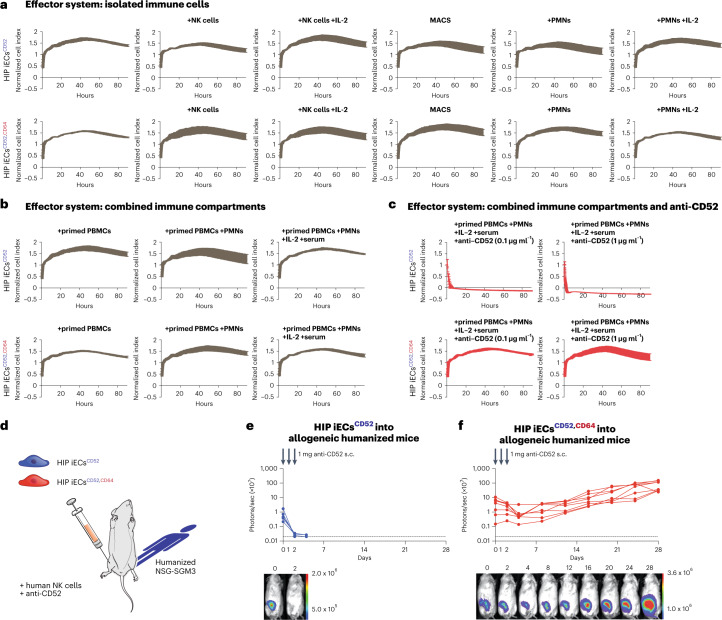
Human HIP iECs^CD52,CD64^ evade all cellular and antibody-mediated immune attacks. **a**, Human HIP iECs^CD52^ and HIP iECs^CD52,CD64^ were challenged with isolated allogeneic innate immune cells. Allogeneic NK cells with or without IL-2, allogeneic macrophages or allogeneic PMN cells with or without IL-2 were used. **b**, Human HIP iECs^CD52^ and HIP iECs^CD52,CD64^ were challenged with primed allogeneic PBMCs after they underwent 10 days of in vitro stimulation with the target cells. Then, PMNs and serum were added to combine all immune compartments of the peripheral blood in this assay. **c**, Human HIP iECs^CD52^ and HIP iECs^CD52,CD64^ were challenged with all immune compartments of the peripheral blood while adding anti-CD52 to enable ADCC and CDC. All graphs show mean ± s.d. and three independent replicates per group and timepoint. **d**, 5 × 10^4^ HIP iECs^CD52^ or HIP iECs^CD52,CD64^ were injected subcutaneously into humanized mice with 10^6^ human NK cells. Both groups received three subcutaneous doses of anti-CD52 IgG1 (alemtuzumab) 1 mg on days 0, 1 and 2. **e**,**f**, BLI signals of HIP iECs^CD52^ (**f**) and HIP iECs^CD52,CD64^ (**g**) were followed (all individual mice were plotted, and BLI pictures of one representative mouse per group are shown). s.c., subcutaneous.

Finally, HIP iECs^CD52,CD64^ were exposed to increasingly harsh allogeneic immune environments to challenge their complete immune-evasiveness. Killing assays were first performed with only allogeneic NK cells, macrophages or polymorphonuclear (PMN) cells (Fig. 4a). Then, the whole cellular immune compartment of peripheral blood mononuclear cells (PBMCs) underwent 10 days of in vitro priming against the target cells to activate any possible adaptive immunity and was then used for the killing assay. We then added two more immune compartments, PMNs and complement-rich serum (Fig. 4b). The fact that the survival of both HIP iECs^CD52^ and HIP iECs^CD52,CD64^ remained unaffected confirms the reliability of the HIP technology to escape all cellular immunity. However, when anti-CD52 was added to enable ADCC and CDC, then HIP iECs^CD52^ were rapidly killed (Fig. 4c). HIP iECs^CD52,CD64^ were resistant against both allogeneic cellular and antibody-mediated killing.

For in vivo testing of complete immune-evasiveness, 5 × 10^4^ HIP iECs^CD52^ or HIP iECs^CD52,CD64^ were injected subcutaneously into humanized NSG-SGM3 mice reconstituted with allogeneic CD34^+^ human hematopoietic stem cells. The cellular grafts were mixed with 1 million human NK cells before injection, and 1-mg doses of alemtuzumab were administered on days 0, 1 and 2 (Fig. 4d). All HIP iECs^CD52^ grafts were quickly rejected (Fig. 4e), whereas all HIP iECs^CD52,CD64^ grafts survived (Fig. 4f). Only HIP iECs^CD52,CD64^ were completely resistant against all forms of allogeneic cellular and antibody-mediated immune attacks.

### Mechanistic features of F_c_ capturing

Macrophages constitutively express CD64 and, thus, have some intrinsic protection against ADCC and CDC in the presence of anti-CD52 (Supplementary Fig. 6). When CD64 was knocked down using short hairpin RNA (shRNA), macrophages lost their protection. To test whether a second antibody would displace anti-CD52 IgG1 from CD64 and break the antibody-evasiveness of HIP iECs^CD52,CD64^, alemtuzumab had to compete with rituximab, a human IgG1 antibody against CD20, an epitope not expressed on iECs. (Supplementary Fig. 7). NK ADCC assays and CDC assays, however, confirmed the robustness of antibody protection, which could not be broken through competitive F_c_ displacement, even when alemtuzumab was given later. When FcγRIIB, the F_c_ receptor with the lowest IgG1 binding affinity, was used instead of CD64, the antibody protection was several orders of magnitudes lower than that for CD64 (Supplementary Fig. 8a–d). In further support of the notion that the protection against antibodies was dependent on the ability of CD64 to capture IgG F_c_, we found that HIP iECs^CD52,CD64^ were resistant against anti-CD52 human IgG1 but susceptible to an anti-CD52 mouse IgG2b, a subclass not binding to CD64^19^ (Supplementary Fig. 8e,f). Antibody protection through CD64 was saturable, with low surface expression failing to protect against higher antibody concentrations (Supplementary Fig. 9a–d). Mechanistically, captured IgG1 bound to and occupied their target epitopes and made them unavailable for antibodies with lower F_c_ affinity to CD64 (Supplementary Fig. 9e), without affecting internalization of antibody–epitope complexes (Supplementary Fig. 9f–h). The next experiments were designed to reveal F_c_ and F_ab_ binding patterns of competitive antibodies. HIP cells were incubated with anti-CD20 IgG1 at fixed dose and increasing doses of anti-CD52 IgG1. When competing for CD64 on HIP iECs^CD64^, anti-CD52 binding increased in a concentration-dependent manner at the expense of anti-CD20 binding (Supplementary Fig. 10a,b). On HIP iECs^CD52,CD64^, the overall binding of anti-CD52 was enhanced and the reduction of anti-CD20 further facilitated with a significant interactive effect that was larger in magnitude (Supplementary Fig. 10c,d). Anti-CD20 showed negligible binding to HIP iECs^CD52,FcγRIIB^ and remained unaffected by anti-CD52 binding (Supplementary Fig. 10e,f), and the interactive effect was not significant. In contrast, when competing with a human IgG1 F_c_ fragment for CD64, the binding of anti-CD20 on HIP iECs^CD64^ and HIP iECs^CD52,CD64^ was qualitatively equivalent (Supplementary Fig. 11). Next, two anti-CD52 antibodies with cytotoxic properties in CDC assays (Supplementary Fig. 12a, Supplementary Fig. 8f and Fig. 3d) were competing for HIP iECs^CD52,CD64^ binding in mouse serum. Only the human IgG1, but not the mouse IgG2b, could additionally ligate CD64. When mainly the mouse IgG2b was bound, we observed rapid target cell killing. When the mouse IgG2b was diminished with increasing concentrations of the human IgG1, the killing gradually slowed down and ceased at the high end (Supplementary Fig. 12b,c). Together, these results show how a favorable binding pattern of cytotoxic antibodies with high F_c_ affinity to CD64 establishes protection even in the presence of competing antibodies.

To assess whether antibody capturing also affects CD8^+^ T cell cytotoxicity, we primed PBMCs from an HLA-A2^−^ donor against HLA-A2^+^ iECs in vitro (Supplementary Fig. 12d). Primed CD8^+^ T cells killed both iECs and iECs^CD64^ with similar kinetics (Supplementary Fig. 12e). However, when cytotoxic anti-HLA-A2 antibodies were added, only iECs were killed more quickly. iECs^CD64^ were protected from CD8^+^ T cell ADCC, even after the target cells were incubated with anti-CD20 for 30 minutes to saturate CD64. Overall, these results confirmed that F_c_ capturing also protects against CD8^+^ T cell ADCC.

Next, applications of this technology were explored. Because CD64 downstream signaling after F_c_ ligation could alter the physiology of some engineered cell types, we used a truncated form of CD64 lacking its intracellular tail for all the following translational applications. Expression of truncated CD64 (CD64t) was found to be similarly effective in preventing NK cell and CD8^+^ T cell ADCC and CDC as full-length CD64 (Supplementary Fig. 13).

### Engineered human thyroid cells evade autoimmune antibody killing

**Fig. 5:**
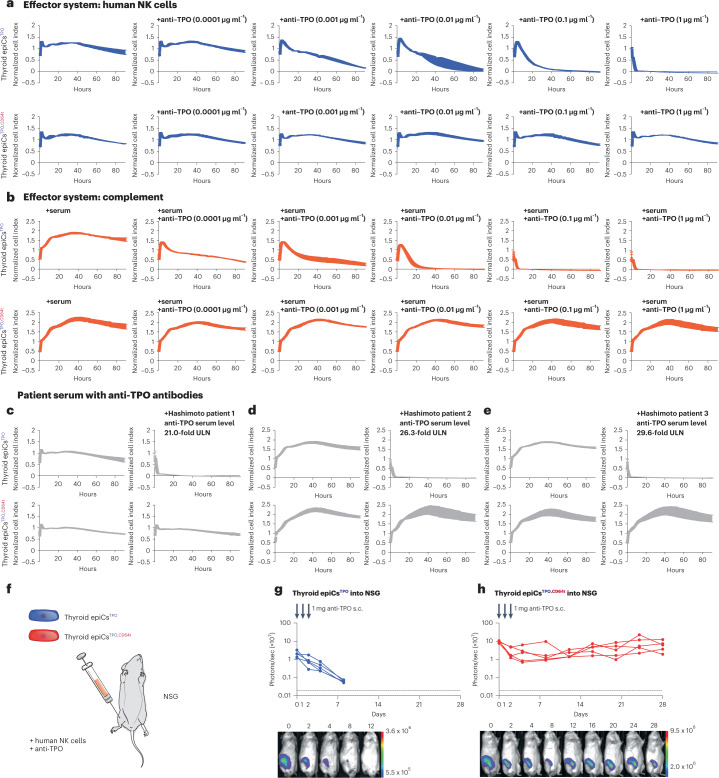
Human thyroid epiCs^TPO,CD64t^ are protected from antibody-mediated killing. **a**,**b**, Human thyroid epiCs^TPO^ and epiCs^TPO,CD64t^ were challenged in impedance NK cell ADCC (**a**) and CDC (**b**) assays with different concentrations of an anti-TPO IgG1 antibody (mean ± s.d.; three independent replicates per group and timepoint). **c**–**e**, Human thyroid epiCs^TPO^ and epiCs^TPO,CD64t^ were incubated with serum from Hashimoto’s patients 1 (**c**), 2 (**d**) and 3 (**e**) in impedance CDC assays (mean ± s.d.; three independent replicates per group and timepoint). ULN, upper limit of normal. **f**, 5 × 10^4^ human thyroid epiCs^TPO^ or epiCs^TPO,CD64t^ were injected subcutaneously into NSG mice with 10^6^ human NK cells. Both groups received three subcutaneous doses of anti-TPO IgG1 1 mg on days 0, 1 and 2. **g**,**h**, BLI signals of thyroid epiCs^TPO^ (**g**) and epiCs^TPO,CD64t^ (**h**) were followed (all individual mice were plotted, and BLI pictures of one representative mouse per group are shown). s.c., subcutaneous.

Hashimoto’s thyroiditis is a prototypic disease in which cytotoxic autoantibodies lead to the destruction of thyroid tissue. Anti-thyroid peroxidase (TPO) antibodies, primarily of IgG1 subclass, are present at high concentrations in 90% of patients^20,21^, have been shown to mediate both ADCC^22–24^ and CDC^25,26^ and diminish the function of the thyroid gland^27^. Thyroid epithelial cells (epiCs) resistant to TPO antibodies would have a much better chance to survive and re-establish organ function if transplanted into hypothyroid patients. Functionally immortalized human thyroid epithelial cells were first transduced to enhance their expression of TPO, because cells in culture tend to downregulate its expression (Supplementary Fig. 14a). Then, epiCs were transduced to express CD64t, which was effective in binding free human IgG1 F_c_ (Supplementary Fig. 14b). Both thyroid epiCs^TPO^ and epiCs^TPO,CD64t^ produced thyroxine irrespective of the presence of IgG1 antibodies (Supplementary Fig. 14c). A humanized anti-TPO IgG1 antibody was effective in killing thyroid epiCs^TPO^ in ADCC and CDC assays, whereas thyroid epiCs^TPO,CD64t^ were fully protected (Fig. 5a,b). Serum samples from three patients with Hashimoto’s thyroiditis and anti-TPO antibody titers 21-fold, 26.3-fold and 29.6-fold the upper level of normal rapidly killed thyroid epiCs^TPO^ in CDC assays (Fig. 5c–e). Thyroid epiCs^TPO,CD64t^ were completely protected from killing in patient serum and, thus, withstood clinically relevant autoimmune conditions. NSG recipients were subcutaneously injected with 5 × 10^4^ epiCs^TPO^ or epiCs^TPO,CD64t^, 1 million human NK cells and 1-mg doses of anti-TPO on days 0, 1 and 2 (Fig. 5f). All epiCs^TPO^ grafts vanished quickly (Fig. 5g), whereas all epiCs^TPO,CD64t^ grafts survived (Fig. 5h).

### Engineered human beta cells evade HLA antibody killing

Type 1 diabetes mellitus (T1DM) is another autoimmune disease, which is T cell mediated with an accompanying antibody response. We aimed to test whether CD64t expression can make them resistant against HLA antibody killing. Human iPSC-derived beta cells transduced to express CD64t (Supplementary Fig. 15a) were able to capture and bind free IgG F_c_ (Supplementary Fig. 15b). Both beta cells and beta cells^CD64t^ showed intact glucose sensing and insulin production irrespective of the presence of antibodies (Supplementary Fig. 15c). HLA-A2-expressing beta cells were killed increasingly quickly with increasing anti-HLA-A2 IgG1 antibody concentrations in ADCC and CDC assays (Supplementary Fig. 15d,e). Beta cells^CD64t^, however, were completely resistant against HLA antibody-mediated killing. We then subcutaneously injected NSG mice with 5 × 10^4^ human beta cells or beta cells^CD64t^ together with 1 million human NK cells. Three 1-mg doses of anti-HLA-A2 IgG1 were subcutaneously injected on days 0, 1 and 2 (Supplementary Fig. 15f). All beta cell grafts vanished within 2 days, whereas all beta cell^CD64t^ grafts defied antibody-mediated killing and paralleled the survival of beta cells without antibody challenge (Supplementary Fig. 15g–i).

### Engineered human CAR T cells evade HLA and non-HLA antibody killing

**Fig. 6:**
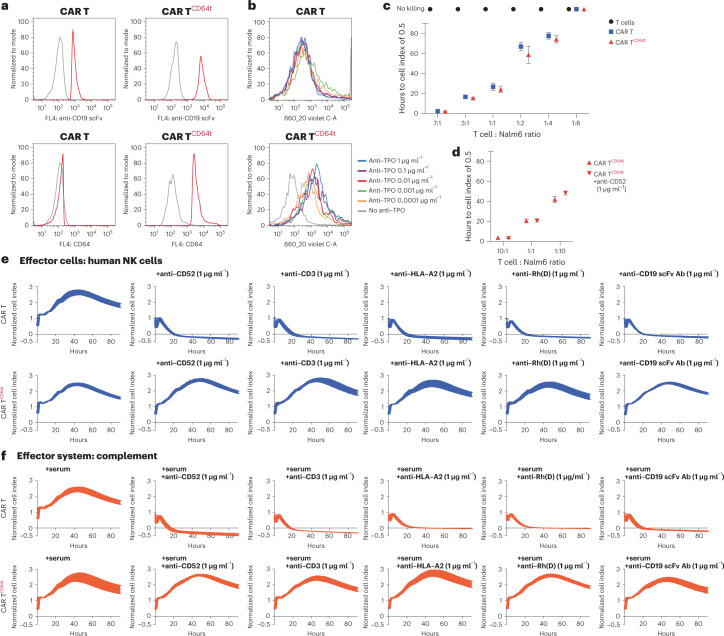
Human CAR T^CD64t^ cells are protected from antibody-mediated killing. **a**, Flow cytometry histograms for anti-CD19 scFv and CD64t expression on human CAR T and CAR T^CD64t^ cells (representative graphs of two independent experiments). **b**, Flow cytometry histograms for the binding of free IgG1 F_c_ (anti-TPO IgG1; representative graph of two independent experiments). **c**, The kinetics of Nalm6 target cell killing by T cells, CAR T cells and CAR T^CD64t^ cells is expressed as hours that it takes for the cell index to drop from 1 to 0.5. Different T cell-to-Nalm6 ratios are shown (mean ± s.d.; three independent replicates per group and timepoint). **d**, The kinetics of Nalm6 target cell killing by CAR T^CD64t^ in the presence and absence of 1 μg ml^−1^ of anti-CD52 is expressed as hours that it takes for the cell index to drop from 1 to 0.5. Different CAR T cell-to-Nalm6 ratios are shown (mean ± s.d.; three independent replicates per group and timepoint). **e**,**f**, Human CAR T and CAR T^CD64t^ cells were challenged in impedance NK cell ADCC (**e**) and CDC (**f**) assays with antibodies against HLA (HLA-A2), non-HLA (CD52 and CD3), rhesus blood type antigen D (Rh(D)) and the CAR (anti-CD19 scFv) at 1 μg ml^−1^ (mean ± s.d.; three independent replicates per group and timepoint).

Clinical CAR T cell therapy induces an antibody response, which is even more pronounced in patients with solid tumors. To test whether we can engineer antibody protection into CAR T cells, we transduced human T cells to express a CD19 scFv-4-1BB-CD3ζ construct with or without additional CD64t expression (Fig. 6a). CAR T^CD64t^ cells were able to capture and bind free IgG1 F_c_ (Fig. 6b). The killing efficacy of CAR T, CAR T^CD64t^ and regular T cells was assessed against CD19^+^ Nalm6 target cells. Whereas control T cells showed no killing, both CAR T and CAR T^CD64t^ cells were equally effective killers across a wide range of effector cell-to-target cell ratios (Fig. 6c). Furthermore, the tumor killing capacity of CAR T^CD64t^ cells was not affected by the presence of F_c_-bound antibodies (Fig. 6d). ADCC and CDC assays with cytotoxic antibodies against an HLA epitope (HLA-A2), non-HLA epitopes (CD52 and CD3), a blood type antigen (Rh(D)) and the CAR receptor (anti-CD19 scFv) were performed, and the CAR T cells were killed in all assays (Fig. 6e,f). CAR T^CD64t^ cells, however, were able to evade antibody-mediated killing with all five antibodies in all assays. CD64t expression does not affect the cytotoxicity of human CAR T cells but makes them resistant against antibodies irrespective of their specificities.

## Discussion

Immune rejection presents the principal hurdle for the success of cell therapeutics, and much effort is currently devoted to developing universal allogeneic off-the-shelf cells that evade cellular rejection^28–30^. Such gene-edited HIP cells, however, remain susceptible to antibody killing directed against non-HLA epitopes, cell-type-specific autoantigens as well as xenogeneic^31^ or synthetic constructs^32^ in engineered cells and viral products from the transduction process^33,34^. Cytotoxic antibodies can be pre-existing or treatment induced^35^ and jeopardize the persistence and efficacy of the cell therapeutics.

We aimed to expand the HIP concept to antibody resistance using non-immunogenic components. Microbial IgG-degrading enzymes have been administered systemically to deplete total IgG and HLA antibodies in highly sensitized patients before kidney transplantation^36^. More recently, these endopeptidases were shown to cleave IgG bound to target cells^37^. However, pre-existing antibodies against these bacterial enzymes are prevalent in the healthy population^38^, spike during streptococcal infections^39,40^ and might themselves add unwanted immunogenicity. We have shown that human CD64 and its truncated form CD64t, which are non-immunogenic, have high affinity for IgG^41^ and protect very effective against antibody killing in several translationally relevant cell types.

Applications for this technology include regenerative cell therapeutics, especially for diseases with an underlying autoimmune component in which antibodies are present that would destroy the transplanted cells^42^. Regenerative cell therapeutics would be destroyed similarly to the native cells if autoimmunity would not be circumvented^42^. Supporting this notion, we show that engineered epiCs^TPO,CD64t^ were protected against clinically relevant anti-TPO killing. The most advanced stem-cell-derived pancreatic islet cells prepared for clinical trials in patients with T1DM are currently produced from embryonic stem cells (ESCs)^43^ and, thus, are transplanted across an HLA mismatch. Currently, such ESC-derived beta cells are transplanted in immunoprotective encapsulation devices^44^ or with immunosuppression^45,46^. We found that forced overexpression of CD64t on human beta cells was sufficient to protect them from anti-HLA ADCC and CDC.

Most success in CAR T cell therapy has been achieved with B cell neoplasms in which patients are treated with lymphocytotoxic drugs, and the CAR T cells directly target the B cell source of antibody production^47^, although antibody induction is still regularly observed^48^. An antibody response against a second infusion of CAR T cells has been documented in four out of 12 patients studied, and none of these patients showed expansion of CAR T cells in their blood^37^. The expression of CD64t on CAR T cells makes them resistant against anti-CAR T cell antibodies without affecting their specific killing capacity. Because early immune clearance is even more common in non-B cell cancer, CAR T^CD64t^ may be more efficient for these indications. The capturing of cytotoxic IgG F_c_ reliably builds protection against antibodies in several cell types and could further advance the immune-evasion concept for allogeneic regenerative and immune-oncology cell therapeutics.

## Methods

### Mice

C57BL/6 (C57BL/6J, B6, H2^b^, 000664), Rag-1 knockout (KO) (B6.129S7-Rag1^tm1Mom^/J, 002216) and NSG (NOD.Cg-*Prkdc*^*scid*^ *Il2rg*^*tm1Wjl*^/SzJ, 005557) mice, 6–12 weeks old, and humanized NSG-SGM3 (NOD.Cg-*Prkdc*^*scid*^ *Il2rg*^*tm1Wjl*^ Tg(CMV-IL3,CSF2,KITLG)1Eav/MloySzJ, 013062) reconstituted with human CD34^+^ hematopoietic stem cells were purchased from The Jackson Laboratory. The number of animals used in the experiments is presented in each figure. Mice received humane care in compliance with the *Guide for the Principles of Laboratory Animals*. Animal experiments were approved by the University of California, San Francisco (UCSF) Institutional Animal Care and Use Committee and performed according to local guidelines.

### Methods for mouse cells

#### Mouse iPSC culture

All iPSCs were grown on mouse embryonic fibroblast (MEF) feeder cells in KO DMEM 10829 with 15% KO Serum Replacement, supplemented with 1% glutamine, 1% MEM-NEAA, 1% penicillin–streptomycin (all Gibco), 0.2% beta-mercaptoethanol and 100 U of leukemia inhibitory factor (LIF) (both Millipore). Medium was changed daily, and the cells were passaged every 2–3 days using 0.05% trypsin-EDTA (Gibco). Cell cultures were regularly screened for mycoplasma infections using the MycoAlert Kit (Lonza). B6 iPSCs were transduced to express Fluc. Then, 100,000 iPSCs were plated in one gelatin-coated six-well plate and incubated overnight at 37 °C in 5% CO_2_. Medium was changed the next day, and one vial of Fluc lentiviral particles expressing luciferase II gene under the re-engineered EF1a promoter (Gentarget) was added. One milliliter of cell medium was added after 36 hours. Then, 24 hours later, complete media change was performed, and, after another 2 days, luciferase expression was confirmed by adding d-luciferin (Promega). Signals were quantified with Spectral Imaging AMI (Spectral Instruments) in maximum photons per second per centimeter square per steradian (p/s/cm^2^/sr). B6 iPSC gene editing to engineer B6 HIP iPSCs was performed as described^6^.

#### EC differentiation of iPSCs

To initiate the differentiation of mouse iPSCs to ECs, medium was changed to RPMI-1640 containing 2% B-27 minus insulin (both Gibco) and 5 μM CHIR-99021 (Selleckchem). Starting on day 2, RPMI-1640 containing 2% B-27 minus insulin (both Gibco) and 2 μM CHIR-99021 (Selleckchem) was used. From day 4 to day 7, cells were exposed to RPMI-1640 EC medium containing 2% B-27 minus insulin plus 50 ng ml^−1^ of mouse vascular endothelial growth factor (mVEGF, R&D Systems), 10 ng ml^−1^ of mouse fibroblast growth factor basic (mFGFb, R&D Systems), 10 μM Y-27632 (Sigma-Aldrich) and 1 μM SB 431542 (Sigma-Aldrich). EC clusters were visible from day 7, and cells were maintained in Endothelial Cell Basal Medium 2 (PromoCell) plus supplements, 10% FCS heat-inactivated (HI) (Gibco), 1% penicillin–streptomycin, 25 ng ml^−1^ of VEGF, 2 ng ml^−1^ of FGFb, 10 μM Y-27632 (Sigma-Aldrich) and 1 μM SB 431542 (Sigma-Aldrich). The differentiation process was completed after 21 days, and undifferentiated cells were detached during the differentiation process. For purification, cells went through MACS purification using anti-CD15 mAb-coated magnetic microbeads (Miltenyi) for negative selection.

#### Transduction and sorting of mouse iECs

In a pre-coated 12-well plate, mouse iECs were plated at a density of 5 × 10^4^ in EC media and then incubated overnight at 37 °C in 5% CO_2_. The next day, cells were incubated overnight at 37 °C in 5% CO_2_ with lentiviral particles carrying a transgene for mouse Cd64 (NM_010186, Gentarget, custom product), human CD64 (NM_000566, Origene, RC207487L2V) or human CD52 (NM_001803, Gentarget, custom product) at a multiplicity of infection (MOI) of 4. Polybrene (8 μg ml^−1^, Millipore) was added to the media, and the plate was centrifuged at 800*g* for 30 minutes before overnight incubation. Cell populations were sorted on a FACSAria (BD Biosciences) for high expression of Cd64, CD64 or CD52 using BV421-labeled anti-mouse Cd64 (FcγRI) antibody (clone X54-5/7.1, BioLegend, 139309, concentration 0.01 mg ml^−1^), BV421-labeled anti-human CD64 antibody (clone 10.1, BD Biosciences, 562872, concentration 0.01 mg ml^−1^) or APC-conjugated anti-human CD52 antibody (clone HI186, BioLegend, 316008, concentration 0.005 mg ml^−1^), respectively.

#### Flow cytometry analysis

For the detection of MHC class I and II surface expression on iECs, cells were stimulated with 100 ng ml^−1^ of IFN-γ and 100 ng ml^−1^ of TNFα (both PeproTech) for 48 hours. Cells were harvested and labeled with flow cytometry antibodies for MHC class I (clone AF6-88.5.5.3, eBioscience, 46-5958-82, concentration 0.01 mg ml^−1^) or mouse IgG2a isotype-matched control antibody (clone eBM2a, eBioscience, 46-4724-80, concentration 0.01 mg ml^−1^), MHC class II (clone M5/114.15.2, eBioscience, 46-5321-82, concentration 0.01 mg ml^−1^) or rat IgG2b isotype-matched control antibody (clone eB149/10H5, eBioscience, 46-4031-80, concentration 0.01 mg ml^−1^), Cd47 (clone miap301, BD Biosciences, 563584, concentration 0.01 mg ml^−1^) or rat IgG2a isotype-matched control antibody (clone R35-95, BD Biosciences, 557690, concentration 0.01 mg ml^−1^). Surface expression of Cd64, CD64 or CD52 was assessed using the antibodies listed above.

To assess mouse Cd64-F_c_ receptor binding, mouse iECs were incubated with different concentrations of mouse IgG2a anti-CD20 (clone rIGEL/773, Abcam, ab219329) and QDot655-labeled F(ab′)2-goat anti-mouse IgG secondary antibody (Thermo Fisher Scientific, Q-11021MP, concentration 0.002 mg ml^−1^). Similarly, human CD64-F_c_ receptor binding was assessed with a humanized IgG1 anti-CD52 (alemtuzumab, ichorbio, ICH4002) and QDot655-labeled F(ab′)2-goat anti-human IgG secondary antibody (Thermo Fisher Scientific, Q-11221MP, concentration 0.002 mg ml^−1^). Cells were analyzed on a LSRFortessa (BD Biosciences), and results were expressed as fold change to isotype-matched control Ig staining. FlowJo 10 was used to analyze flow cytometric data.

#### Mouse NK cell isolation

C57BL/6 mice were stimulated with poly I:C injections (100 μg intraperitoneally, Sigma-Aldrich) and, 18 hours later, NK cells were isolated from spleens. After red blood cell lysis, NK cells were purified using the MagniSort Mouse NK Cell Enrichment Kit (Invitrogen), followed by CD49b MACS-sorting (Miltenyi). This mouse NK cell population had a purity of >99%.

#### XCelligence killing assays

Real-time killing assays were performed on the XCelligence SP platform and MP platform (ACEA Biosciences). Special 96-well E-plates (ACEA Biosciences) coated with collagen (Sigma-Aldrich) were used. A total of 4 × 10^4^ mouse iECs were plated in 100 μl of medium. After the cell index reached 0.7, we added 4 × 10^4^ C57BL/6 6 NK cells to ADCC assays or 50 μl of C57BL/6 serum to CDC assays. The following antibodies were used and added after mixing with the NK cells in 50 μl of medium or in the serum as indicated: mouse IgG2a anti-H-2^b^ (BioXCell, clone AF6-88.5.5.3, BE0121) and humanized anti-CD52 IgG1 (alemtuzumab, ichorbio, ICH4002). Different concentrations ranging from 0.0001 μg ml^−1^ to 1 μg ml^−1^ were used. As a negative control, cells were treated with 2% Triton X-100 in medium. Data were standardized and analyzed with RTCA Pro 2.3.2 software (ACEA Biosciences).

#### Survival analysis of mouse iECs using BLI

One million firefly Luc^+^ B6 HIP iECs^CD52^ and B6 HIP iECs^CD52,CD64^ were transplanted subcutaneously, and 1 mg of alemtuzumab was injected into the peritoneum on post-transplant days 0 and 3. For imaging, d-luciferin firefly potassium salt (375 mg kg^−1^, Biosynth) was dissolved in PBS (pH 7.4, Gibco), and 250 μl was injected intraperitoneally in anesthetized mice. Animals were imaged in the AMI HT (Spectral Instruments). Region of interest (ROI) bioluminescence was quantified in units of maximum p/s/cm^2^/sr. The maximum signal from an ROI was measured using Aura Imaging software (Spectral Instruments).

### Methods for human cells

#### Human iPSC culture

Human iPSCs were cultured on diluted feeder-free Matrigel-coated 10-cm dishes (hESC qualified, BD Biosciences) in Essential 8 Flex Medium (Thermo Fisher Scientific). Medium was changed every 24 hours, and Versene (Gibco) was used for cell passaging at a ratio of 1:6. For luciferase transduction, 1 × 10^5^ iPSCs were plated in one six-well plate and grown overnight at 37 °C in 5% CO_2_. Medium was changed the next day, and 200 μl of Fluc lentiviral particles expressing luciferase II gene under re-engineered EF1a promotor (Gentarget) was added. After 36 hours, 1 ml of cell medium was added. After another 24 hours, complete medium change was performed. Then, 2 days later, luciferase expression was confirmed by adding d-luciferin (Promega). Signals were quantified in p/s/cm^2^/sr. Human iPSC gene editing to engineer HIP iPSCs was performed as described^6^.

#### Endothelial cell differentiation from human iPSCs

When the iPSC confluency reached 60%, the differentiation was initiated, and medium was changed to RPMI-1640 containing 2% B-27 minus insulin (both Gibco) and 5 μM CHIR-99021 (Selleckchem). On day 2, the medium was changed to RPMI-1640 containing 2% B-27 minus insulin (Gibco) and 2 μM CHIR-99021 (Selleckchem). From culture day 4 to day 7, cells were exposed to RPMI-1640 EC medium, RPMI-1640 containing 2% B-27 minus insulin plus 50 ng ml^−1^ of VEGF (PeproTech), 10 ng ml^−1^ of human FGFb (PeproTech), 10 μM Y-27632 (Sigma-Aldrich) and 1 μM SB 431542 (Sigma-Aldrich). EC clusters were visible from day 7, and cells were maintained in Endothelial Cell Basal Medium 2 (PromoCell) plus supplements, 10% FCS HI (Gibco), 1% penicillin–streptomycin, 25 ng ml^−1^ of VEGF, 2 ng ml^−1^ of FGFb, 10 μM Y-27632 (Sigma-Aldrich) and 1 μM SB 431542 (Sigma-Aldrich). The differentiation protocol was completed after 14 days, and undifferentiated cells were detached during the differentiation process. TrypLE (Gibco) was used for passaging the cells 1:3 every 3–4 days.

#### NK cell culture

Human primary NK cells were purchased from STEMCELL Technologies (70036) and cultured in RPMI-1640 plus 10% FCS HI and 1% penicillin–streptomycin before performing the assays.

#### Macrophage differentiation from PBMCs

PBMCs were isolated by Ficoll separation from fresh blood and re-suspended in RPMI-1640 with 10% FCS HI and 1% penicillin–streptomycin (all Gibco). Cells were plated in 24-well plates at a concentration of 1 × 10^6^ cells per milliliter, and 10 ng ml^−1^ of human M-CSF (PeproTech) was added to the medium. Medium was changed every other day. From day 6 onward, 1 μg ml^−1^ of human IL-2 (PeproTech) was added to the medium for 24 hours before performing assays.

#### Human thyroid epithelial cells

Immortalized human thyroid epiCs were purchased from InSCREENex (INS-CI-1017) and cultured in the media provided from the manufacturer (INS-ME-1017, InSCREENex). The cells were maintained in culture and passaged using TryplE 1:3 every 3–4 days.

#### Human pancreatic beta cells

Human iPSC-derived pancreatic beta cells were purchased from Takara Bio (ChiPSC22, Y10106) and cultured in Cellartis hiPS Beta Cell Media Kit (Takara Bio, Y10108). Cells were plated in 12-well plates according to the manufacturerʼs protocol. Some cells were transduced with CD64t lentiviral particles (Gentarget).

#### Human CAR T cells

Human anti-CD19 CAR T cells were generated from human PBMCs using lentiviral particles carrying a transgene for the CD19 scFv-4-1BB-CD3ζ construct (ProMab, PM-CAR1002-V). PBMCs were stimulated with IL-2 overnight and seeded in 96-well U-bottom plates at a density of 10^5^ cells per well containing protamine sulfate and 0.1 μg ml^−1^ of IL-2 (PeproTech). Lentiviruses were added to the wells at an MOI of 20. Some wells were transduced additionally with CD64t lentiviral particles at an MOI of 20 (Gentarget). Spinfection was carried out at 1,800 r.p.m. for 30 minutes at 25 °C. After that, the cells were returned to a humidified 5% CO_2_ incubator overnight. Medium was changed after 2 days, and cells were seeded at a density of 10^6^ per milliliter in T cell media (OpTmizer, Thermo Fisher Scientific). CD3/CD28 beads (Thermo Fisher Scientific) were used for T cell expansion. Cells were sorted for CAR^+^ and CAR^+^/CD64t^+^ populations on a BD FACSAria Fusion and used for further assays.

#### Transduction and sorting of human cells

In a pre-coated 12-well plate, human iECs, thyroid epiCs or beta cells were plated at a density of 5 × 10^4^ in cell-specific media and then incubated overnight at 37 °C in 5% CO_2_. The next day, cells were incubated overnight at 37 °C in 5% CO_2_ with lentiviral particles carrying a transgene for human CD52 (NM_001803, Gentarget, custom product), human CD64 (NM_000566, Origene, RC207487L2V), human TPO (NM_000547, Gentarget, custom product) or a truncated form of human CD64 (CD64t, ATGTGGTTCTTGACAACTCTGCTCCTTTGGGTTCCAGTTGATGGGCAAGTGGACACCACAAAGGCAGTGATCACTTTGCAGCCTCCATGGGTCAGCGTGTTCCAAGAGGAAACCGTAACCTTGCACTGTGAGGTGCTCCATCTGCCTGGGAGCAGCTCTACACAGTGGTTTCTCAATGGCACAGCCACTCAGACCTCGACCCCCAGCTACAGAATCACCTCTGCCAGTGTCAATGACAGTGGTGAATACAGGTGCCAGAGAGGTCTCTCAGGGCGAAGTGACCCCATACAGCTGGAAATCCACAGAGGCTGGCTACTACTGCAGGTCTCCAGCAGAGTCTTCACGGAAGGAGAACCTCTGGCCTTGAGGTGTCATGCGTGGAAGGATAAGCTGGTGTACAATGTGCTTTACTATCGAAATGGCAAAGCCTTTAAGTTTTTCCACTGGAATTCTAACCTCACCATTCTGAAAACCAACATAAGTCACAATGGCACCTACCATTGCTCAGGCATGGGAAAGCATCGCTACACATCAGCAGGAATATCTGTCACTGTGAAAGAGCTATTTCCAGCTCCAGTGCTGAATGCATCTGTGACATCCCCACTCCTGGAGGGGAATCTGGTCACCCTGAGCTGTGAAACAAAGTTGCTCTTGCAGAGGCCTGGTTTGCAGCTTTACTTCTCCTTCTACATGGGCAGCAAGACCCTGCGAGGCAGGAACACATCCTCTGAATACCAAATACTAACTGCTAGAAGAGAAGACTCTGGGTTATACTGGTGCGAGGCTGCCACAGAGGATGGAAATGTCCTTAAGCGCAGCCCTGAGTTGGAGCTTCAAGTGCTTGGCCTCCAGTTACCAACTCCTGTCTGGTTTCATGTCCTTTTCTATCTGGCAGTGGGAATAATGTTTTTAGTGAACACTGTTCTCTGGGTGACAATATAG, Gentarget, custom product) at an MOI of 4. Polybrene (8 μg ml^−1^, Millipore) was added to the media, and the plate was centrifuged at 800*g* for 30 minutes before overnight incubation. Cell populations were sorted on a FACSAria (BD Biosciences) using APC-conjugated anti-human CD52 antibody (clone HI186, BioLegend, 316008, concentration 0.005 mg ml^−1^), BV421-labeled anti-human CD64 antibody (clone 10.1, BD Biosciences, 562872, concentration 0.01 mg ml^−1^), PE-conjugated anti-human TPO antibody (clone MoAb47, Santa Cruz Biotechnology, sc-58432, concentration 0.01 mg ml^−1^) or FITC-conjugated anti-FMC63 scFv (clone Y45, Acro Biosystems, FM3-FY45, dilution 1:50).

#### CD64 knockdown in macrophages

Human peripheral blood macrophages were purchased from STEMCELL Technologies (70042) and seeded in 12-well plates at a density of 1 × 10^5^ cells per well in RPMI-1640 media (Gibco) supplemented with 10% FCS HI (Sigma Aldrich). The next day, lentiviral particles with shRNA for human CD64 (custom order, Gentarget) were added to the media at an MOI 20, and 1 μg ml^−1^ of protamine sulfate was added. After 72 hours, the macrophages were harvested and sorted for CD64 deficiency using a BV421 mouse anti-human CD64 antibody (clone 10.1, 562872, BD Biosciences, concentraztion 0.01 mg ml^−1^) and the isotype-matched control mouse IgG1κ antibody (clone MOPC-21, 400157, BioLegend, concentration 0.01 mg ml^−1^) on a FACSAria flow cytometer (BD Biosciences).

#### FcγRIIB expression in HIP iECs

A six-well plate was coated with gelatin, and 1 × 10^5^ HIP iECs^CD52^ per well were seeded in 2.5 ml of EC media and incubated for 24 hours at 37 °C in 5% CO_2_. The next day, 1 ml of fresh EC media was added to the wells, and human FcγRIIB lentiviral particles (custom order, Gentarget) were added to the media at an MOI of 20, and 1 μg ml^−1^ of protamine sulfate was also added. After 24 hours, 1 ml of EC media was added to the cells, and, after subsequent 48 hours, a complete media change was performed. The cells were harvested and stained for FcγRIIB expression using an APC mouse anti-human CD32B/C antibody (clone S18005H, 398304, BioLegend, concentration 0.0025 mg ml^−1^) together with the isotype-matched control APC mouse IgG1κ antibody (clone MOPC-21, 555751, BD Biosciences, concentration 0.0025 mg ml^−1^). The FcγrIIB^+^ cells were sorted on a FACSAria flow cytometer (BD Biosciences).

#### Flow cytometry analysis

Human iPSCs were grown in six-well plates in medium containing 100 ng ml^−1^ of IFN-γ and 100 ng ml^−1^ of TNFα for 48 hours. Cells were harvested and labeled with APC-conjugated anti-HLA-A,B,C antibody (clone G46_2.6, BD Biosciences, 562006, concentration 0.05 mg ml^−1^) for HLA class I detection or APC-conjugated IgG1 isotype-matched control antibody (clone MOPC-21, BD Biosciences, 555751, concentration 0.05 mg ml^−1^). For HLA class II detection, cell were labeled with Alexa Fluor 647-labeled anti-HLA-DR,DP,DQ antibody (clone Tu39, BD Biosciences, 563591, concentration 0.01 mg ml^−1^) or Alexa Fluor 647-labeled IgG2a isotype-matched control antibody (clone G155-178, BD Biosciences, 557715, concentration 0.01 mg ml^−1^). PerCP-Cy5.5-conjugated anti-CD47 (clone B6H12, BD Biosciences, 561261, concentration 0.01 mg ml^−1^) or PerCP-Cy5.5-conjugated IgG1 isotype-matched control antibody (clone MOPC-21, BD Biosciences, 552834, concentration 0.01 mg ml^−1^) were used for detection of CD47. For MICA detection, a humanized anti-MICA IgG1 (Creative Biolabs, TAB-0799CL, concentration 0.01 mg ml^−1^) with PE-labeled mouse anti-human IgG1 F_c_ secondary antibody (clone HP6001, SouthernBiotech, 9054-09, concentration 0.01 mg ml^−1^) was used. For SIRPα detection, an APC-labeled mouse anti-human SIRPα (clone 15-414, 372106, BioLegend, concentration 0.02 mg ml^−1^) with IgG2aκ isotype-matched control antibody (557715, BD Biosciences, concentration 0.02 mg ml^−1^) was used. Surface expression of CD52, CD64/CD64t, TPO and anti-CD19 scFv was assessed using the antibodies listed above. To study competing CD52 binding, an APC-conjugated anti-CD52 mouse IgG2b was used (clone HI186, GTX80134, GeneTex).

To assess human CD64-F_c_ binding, a humanized IgG1 anti-CD52 (alemtuzumab, ichorbio, ICH4002), a humanized IgG1 anti-TPO (clone B8, Creative Biolabs, FAMAB-0014JF) or humanized anti-SIRPα antibodies (clone KWAR23, IgG1, IgG2, IgG3 or IgG4, custom order, Creative Biolabs) and QDot655-labeled F(ab′)2-goat anti-human IgG secondary antibody (Thermo Fisher Scientific, Q-11221MP, 0.002 mg ml^−1^) were used.

To assess alemtuzumab internalization, cells were incubated with 1 μg ml^−1^ of Alexa Fluor 488-conjugated anti-CD52 (clone Hu116, FAB9889G, R&D Systems) for different time periods. Then, an anti-Alexa Fluor 488 quenching antibody (Invitrogen, A-11094) was used at 10 μg ml^−1^ for 30 minutes. Flow cytometry of cells with and without the quenching antibody was done to assess total and intracellular fluorescence, respectively. Cells were analyzed on a LSRFortessa (BD Biosciences), and results were expressed as fold change to isotype-matched control Ig staining.

#### XCelligence killing assays

Real-time killing assays were performed on the XCelligence SP platform and MP platform (ACEA Biosciences). Special 96-well E-plates (ACEA Biosciences) coated with gelatin (Sigma-Aldrich) were used. A total of 4 × 10^4^ human iECs, epiCs, beta cells, CAR T cells or macrophages were plated in 100 μl of medium. After the cell index reached 0.7, we added 4 × 10^4^ human NK cells to ADCC assays or 50 μl of blood-type-compatible human serum to CDC assays. Patient serum samples were treated with DTT to eliminate blood type IgM antibodies before use. For NALM killing assays, 4 × 10^4^ NALM cells were plated, and 4 × 10^4^ CAR T cells were used as effector cells. The following antibodies were used and added after mixing with the NK cells in 50 μl of medium or in the serum as indicated: humanized anti-CD52 IgG1 (alemtuzumab, ichorbio, ICH4002), humanized anti-MICA IgG1 (Creative Biolabs, TAB-0799CL), humanized anti-HLA-A2 IgG1 (clone 3PF12, Absolute Antibody, AB00947-10.0), humanized anti-Rh(D) IgG1 (clone F5, Creative Biolabs, FAMAB-0089WJ), humanized anti-TPO IgG1 (clone B8, Creative Biolabs, FAMAB-0014JF), humanized anti-CD3 IgG1 (Creative Biolabs, custom product) and humanized anti-CD19 scFv (FMC63) IgG1 (clone 136.20.1, Creative Biolabs, HPAB-0440-YJ-m/h). Different concentrations ranging from 0.0001 μg ml^−1^ to 1 μg ml^−1^ were used. As a negative control, cells were treated with 2% Triton X-100 in medium (data not shown). Data were standardized and analyzed with RTCA software (ACEA Biosciences).

#### Ex vivo T cell priming

Blood from an HLA-A2^−^ donor was collected, and PBMCs were obtained after Ficoll separation. The PBMCs were primed by co-culturing 5 × 10^5^ HLA-A2^+^ wild-type iEC cells and 1 × 10^6^ PBMCs in gelatin-coated flasks. The media, which consisted of a 1:1 mixture of EC medium and PBMC medium, was changed every 3 days. After 14 days, the cells in suspension were harvested and sorted using an APC mouse anti-human CD3 antibody (clone SP34-2, 557597, BD Biosciences, concentration 0.01 mg ml^−1^) together with the isotype-matched control APC mouse IgG1κ antibody (clone MOPC-21, 550854, BD Biosciences, concentration 0.01 mg ml^−1^) and a BV421 mouse anti-human CD8 antibody (clone SK1, 344748, BioLegend, concentration 0.005 mg ml^−1^) together with the isotype-matched control mouse IgG1κ antibody (clone MOPC-21, 400157, BioLegend, concentration 0.005 mg ml^−1^). The CD3^+^CD8^+^ cells were sorted using a FACSAria flow cytometer (BD Biosciences) and used for real-time XCelligence killing assays with the anti-HLA-A2 IgG1 antibody, with or without prior treatment with anti-CD20 IgG1.

#### Survival analysis of human iECs using BLI

A total of 5 × 10^4^ HIP iECs^CD52^ or HIP iECs^CD52,CD64^ were injected subcutaneously into NSG mice or humanized NSG-SGM3 mice mixed together with 1 million human NK cells with or without 1 mg of alemtuzumab. On the two subsequent days, 1-mg doses of alemtuzumab were injected subcutaneously into the vicinity of the cell transplants where indicated. Transplant experiments with human thyroid epiCs^TPO^ and epiCs^TPO,CD64t^ or human beta cells and beta cells^CD64t^ were similarly performed in NSG mice with anti-TPO IgG1 or anti-HLA-A2 IgG1, respectively. All injected cells were Luc^+^. For imaging, d-luciferin firefly potassium salt (375 mg kg^−1^, Biosynth) was dissolved in PBS (pH 7.4, Gibco), and 250 μl was injected intraperitoneally in anesthetized mice. Animals were imaged in the AMI HT (Spectral Instruments). ROI bioluminescence was quantified in units of maximum p/s/cm^2^/sr. The maximum signal from an ROI was measured using Aura Imaging software (Spectral Instruments).

#### In vivo cytotoxicity assay with adoptive transfer

Five million DiO-labeled HIP iECs^CD52^ and 5 million DiD-labeled HIP iECs^CD52,CD64^ were mixed and injected intraperitoneally into NSG mice (Vybrant Multicolor Cell-Labeling Kit, Thermo Fisher Scientific). A total of 10^8^ IL-2-stimulated human primary NK cells (STEMCELL Technologies) or 10^8^ macrophages (differentiated from PBMCs) were also intraperitoneally injected. Human primary NK cells were pre-treated with human IL-2 (1 μg ml^−1^, PeproTech) for 12 hours before injection. After 48 hours, cells were collected from the peritoneum, and the ratio of both cell populations was assessed by flow cytometry (FACSCalibur, BD Biosciences).

#### Thyroxine ELISA

A 96-well plate was coated with gelatin, and 3 × 10^4^ human thyroid epiCs^TPO^ or epiCs^TPO,CD64t^ per well were seeded in 100 μl of h7H media^49^ and incubated for 24 hours at 37 °C in 5% CO_2_. The next day, the h7H media was changed and supplemented with 1 mU ml^−1^ of native bovine thyroid-stimulating hormone (TSH) protein (TSH-1315B, Creative BioMart). Three wells per epiC group were also supplemented with 1 μg ml^−1^ of anti-CD52 IgG1 (alemtuzumab, clone Campath-1H, Bio-Rad). After 72 hours, the supernatant was collected, and the level of thyroxine was assessed using the thyroxine (T4) competitive ELISA kit (EIAT4C, Invitrogen) according to the manufacturer’s instructions. Results are presented as change in optical density (OD) between groups with and without alemtuzumab.

#### Insulin ELISA

A 24-well plate was coated with gelatin, and 5 × 10^4^ iPSC-derived beta cells and beta cells^CD64t^ (Y10108, Takara Bio) per well were seeded in 500 μl of Cellartis hiPS beta cell media and incubated for 24 hours at 37 °C in 5% CO_2_. The next day, the Cellartis hiPS beta cell media was changed to RPMI-1640 without glucose (11879-020, Gibco) for 2 hours. After 2 hours, the media was changed to RPMI without glucose supplemented with 2 mM glucose (G7528, Sigma-Aldrich). Three wells per beta cell group were also supplemented with 1 μg ml^−1^ of anti-CD52 IgG1 (alemtuzumab, clone Campath-1H, MCA6101, Bio-Rad). After 20 minutes, the supernatant was collected, and the media was changed to RPMI without glucose supplemented with 20 mM glucose. Again, 1 μg ml^−1^ of alemtuzumab was added to three wells per group. After 20 minutes, the supernatant was collected, and the level of human insulin was determined using the human insulin ELISA kit (KAQ1251, Invitrogen) according to the manufacturer’s instructions. Results are presented as change in OD between groups with and without alemtuzumab.

### Statistics

All data are expressed as mean ± s.d. Intergroup differences were appropriately assessed by either unpaired Student’s *t*-test or one-way ANOVA with Bonferroni’s post hoc test. To determine if the effect of anti-CD52 concentration on fluorescence intensity differed significantly by antibody type, linear regression was performed with mean fluorescence intensity as the dependent variable and an interaction term for antibody type (anti-CD52 versus anti-CD20) by ten-fold increase units of anti-CD52 concentration (μg ml^−1^). Hypothesis tests were two-sided, and the significance threshold was set to 0.05. To determine if the cell lines differed significantly in their capacity to internalize alemtuzumab, linear regression was performed with percentage of internalized alemtuzumab as the dependent variable and an interaction term for cell line (hiECs^CD52^ versus hiECs^CD52,CD64^) by time after exposure to alemtuzumab. Exposure to alemtuzumab was treated as a continuous variable (1 = 10 minutes, 2 = 2 hours, 3 = 24 hours). Hypothesis tests were two-sided, and the significance threshold was set to 0.05. Statistical analyses were performed using SAS version 9.4.

### Reporting summary

Further information on research design is available in the Nature Research Reporting Summary linked to this article.

## Online content

Any methods, additional references, Nature Research reporting summaries, source data, extended data, supplementary information, acknowledgements, peer review information; details of author contributions and competing interests; and statements of data and code availability are available at 10.1038/s41587-022-01540-7.

## Supplementary information

Supplementary Information Supplementary Figs. 1–15

Reporting Summary

## Data Availability

All data generated or analyzed during this study are included in this published article and its Supplementary Information files. No pre-established data exclusion method was used. No clinical data were included. Supplementary Information is available in the online version of the paper.
