# Supplementary material for: Protection of cell therapeutics from antibody-mediated killing by CD64 overexpression
Source: Nat Biotechnol. 2023 Jan 2;41(5):717–27. doi: 10.1038/s41587-022-01540-7 (PMC10188358; doi:10.1038/s41587-022-01540-7)
Supplement: Supplementary file 1 — Supplementary Figs. 1–15 [file 41587_2022_1540_MOESM1_ESM.pdf]

nature biotechnology

Article

https://doi.org/10.1038/s41587-022-01540-7

# Protection of cell therapeutics from antibody-mediated killing by CD64 overexpression

In the format provided by the authors and unedited

**Supplementary Information**

**Table of contents**

**Supplementary Figure 1**: *Mouse Cd64 protects B6 iECs from MHC antibody-mediated killing*.

**Supplementary Figure 2**: *CD64 expression protects B6 HIP iECs$^{CD52,CD64}$ from non-MHC antibody killing in vitro and in vivo.*

**Supplementary Figure 3**: *CD64 expression binds IgG isotypes and protects human iECs from non-HLA antibody killing.*

**Supplementary Figure 4**: *CD64 expression protects human HIP iECs from rhesus blood type antibody killing.*

**Supplementary Figure 5**: *CD64 expression protects human HIP iECs$^{CD52,CD64}$ from non-HLA antibody NK cell and macrophage killing in vivo*.

**Supplementary Figure 6**: *CD64-knockdown macrophages have increased susceptibility for ADCC and CDC killing.*

**Supplementary Figure 7**: *The $F_c$ capture mechanism works despite the presence of competing antibodies and serum.*

**Supplementary Figure 8**: *High-affinity IgG $F_c$ receptor binding is necessary to establish antibody protection.*

**Supplementary Figure 9**: *Antibody protection depends on expression levels of CD64 and involves occupation of target antigens.*

**Supplementary Figure 10**: *Competitive anti-CD20 vs. anti-CD52 binding.*

**Supplementary Figure 11**: *Competitive anti-CD20 vs. human IgG1 $F_c$ fragment.*

**Supplementary Figure 12**: *CD64 protects from CDC with competitive antibodies and from $CD8^+$ T cell ADCC.*

**Supplementary Figure 13**: *Truncated CD64 similarly protects from antibody-mediated killing*.

**Supplementary Figure 14**: *Characterization of human thyroid epiCs$^{TPO,CD64t}$*.

**Supplementary Figure 15**: *Human beta cells$^{CD64t}$ are protected from antibody-mediated killing.*

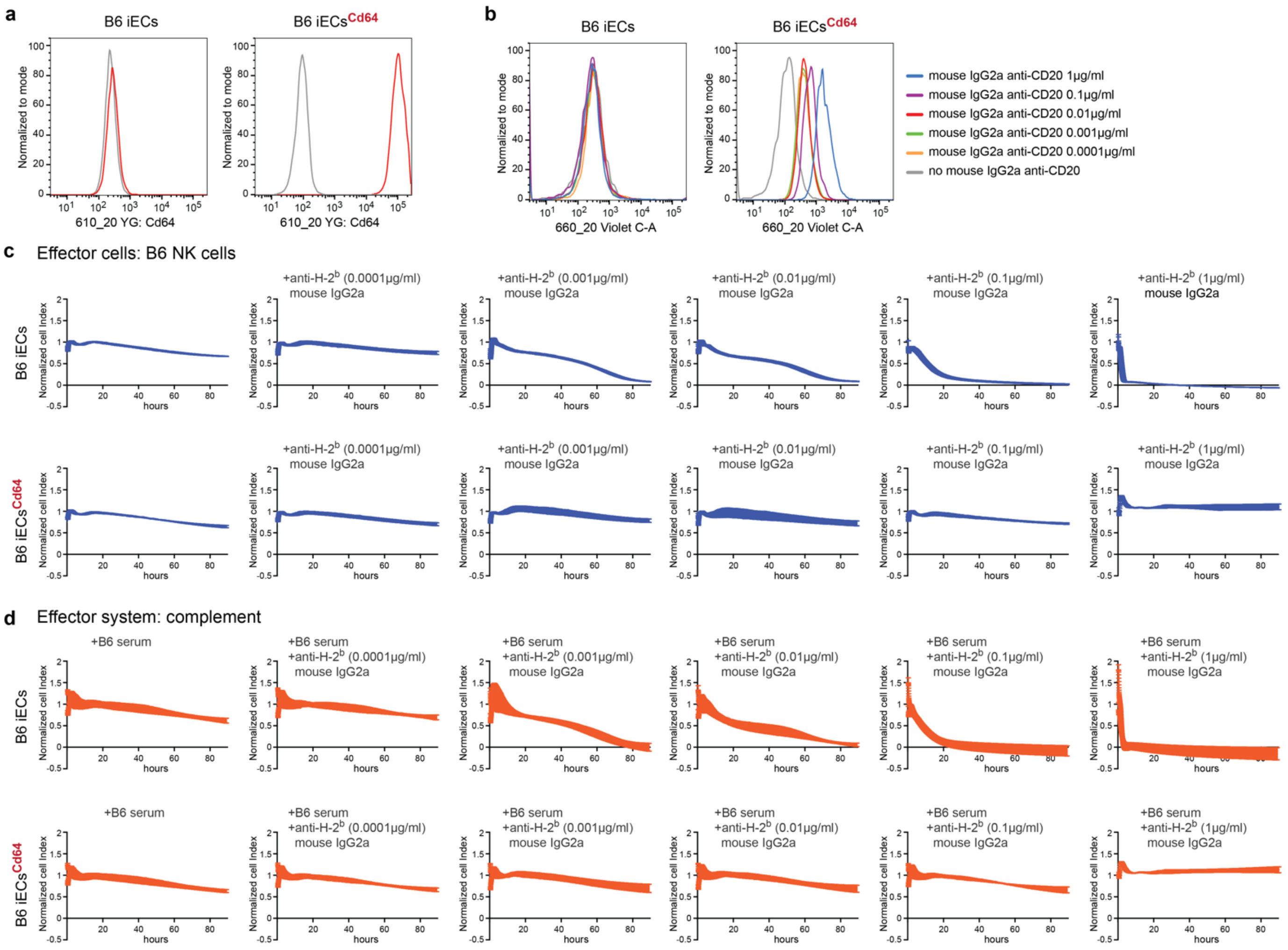


**Supplementary Figure 1**: *Mouse Cd64 protects B6 iECs from MHC antibody-mediated killing*. **a**, Flow cytometry histograms for Cd64 expression on B6 iECs and B6 iECs$^{Cd64}$ (representative graphs of two independent experiments). **b**, Flow cytometry histograms for the binding of free mouse IgG2a $F_c$ (anti-CD20, representative graph of two independent experiments). **c-d**, B6 iECs and B6 iECs$^{Cd64}$ were challenged in impedance NK cell ADCC (c) and CDC (d) assays with different concentrations of an anti-H-2$^b$ mouse IgG2a antibody (mean ± SD, three independent replicates per group and time point).

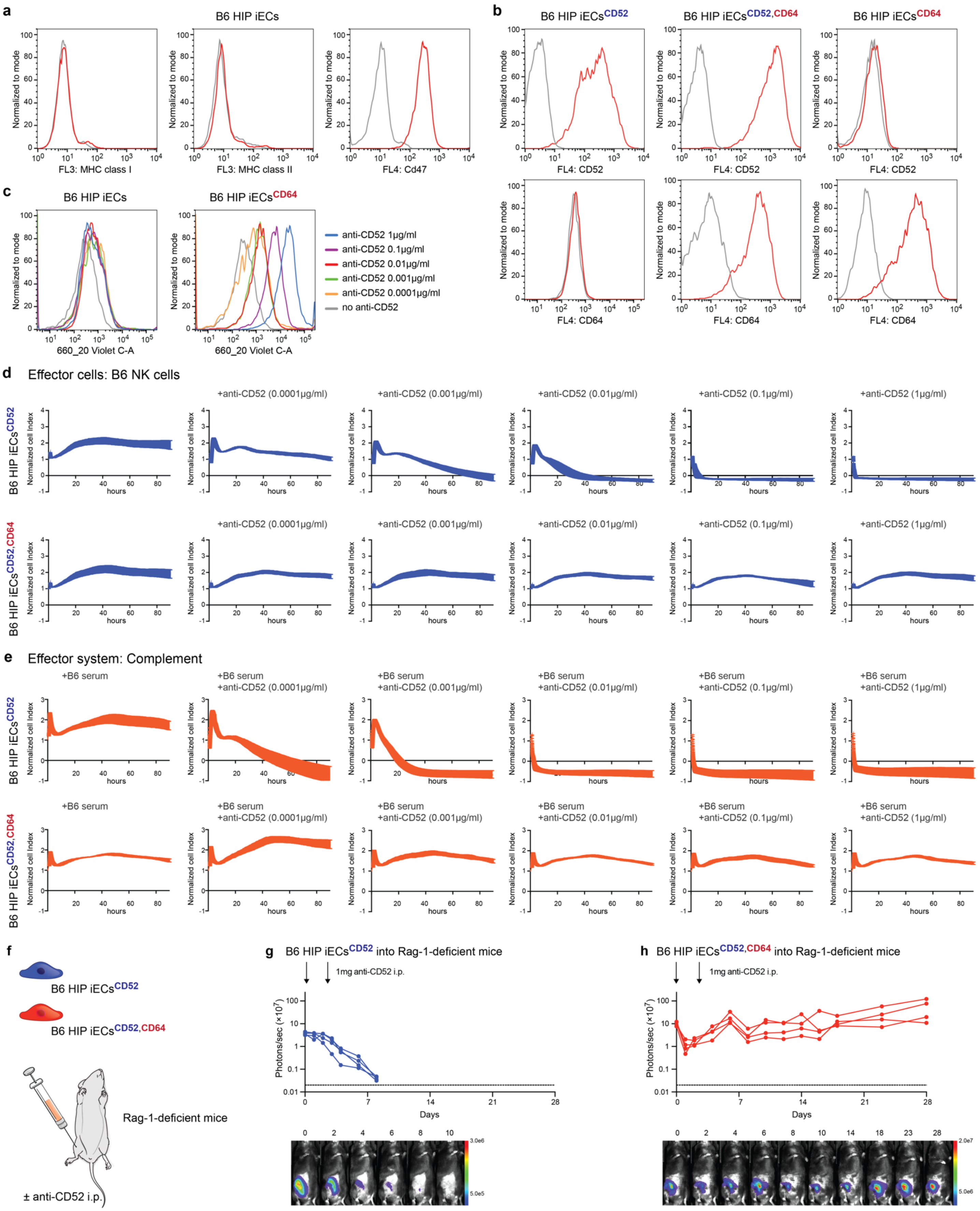


**Supplementary Figure 2**: *CD64 expression protects B6 HIP iECs$^{CD52,CD64}$ from non-MHC antibody killing in vitro and in vivo.* **a**, The HIP immune phenotype of MHC class I and II deficiency and Cd47 overexpression in

B6 HIP was confirmed in flow cytometry (representative graph of two independent experiments). **b**, Flow cytometry histograms for CD52 and CD64 expression on B6 HIP iECs$^{CD52}$, B6 HIP iECs$^{CD52,CD64}$, and B6 HIP iECs$^{CD64}$ (representative graphs of two independent experiments). **c**, Flow cytometry histograms for the binding of free IgG1 $F_c$ (anti-CD52, alemtuzumab, representative graph of two independent experiments). **d-e**, B6 HIP iECs$^{CD52}$ and B6 HIP iECs$^{CD52,CD64}$ were challenged in impedance NK cell ADCC (d) and CDC (e) assays with different concentrations of an anti-CD52 IgG1 antibody (mean ± SD, three independent replicates per group and time point). **f**, B6 HIP iECs$^{CD52}$ and B6 HIP iECs$^{CD52,CD64}$ were subcutaneously injected into Rag-1-deficient mice. Alemtuzumab (anti-CD52) was administered intraperitoneally on day 0 and 3 and cell survival was followed. **g-h**, B6 HIP iECs$^{CD52}$ (g) and B6 HIP iECs$^{CD52,CD64}$ (h) BLI signals were followed (all individual mice were plotted and BLI pictures of one representative mouse per group are shown).

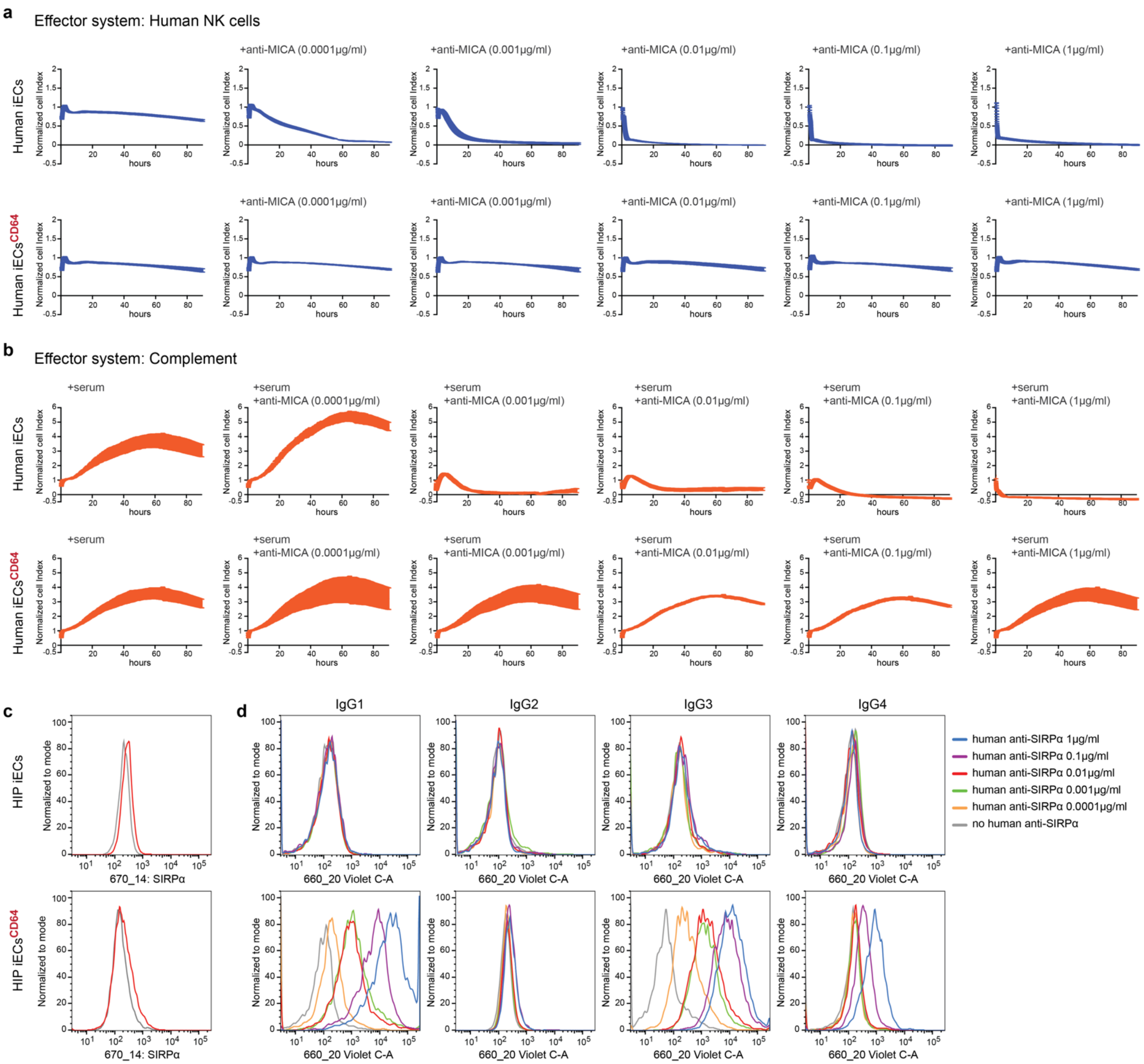


**Supplementary Figure 3**: *CD64 expression binds IgG isotypes and protects human iECs from non-HLA antibody killing.* **a-b**, Human iECs and iECs$^{CD64}$ were challenged in impedance NK cell ADCC (a) and CDC (b) assays with different concentrations of an anti-MICA IgG1 antibody (mean ± SD, three independent replicates per group and time point). **c**, Flow cytometry histograms for SIRP$\alpha$ expression on HIP iECs and HIPiECs$^{Cd64}$ (representative graphs of two independent experiments). **d**, Flow cytometry histograms for the binding of anti-SIRP$\alpha$ IgG1, IgG2, IgG3, and IgG4 (representative graph of two independent experiments).

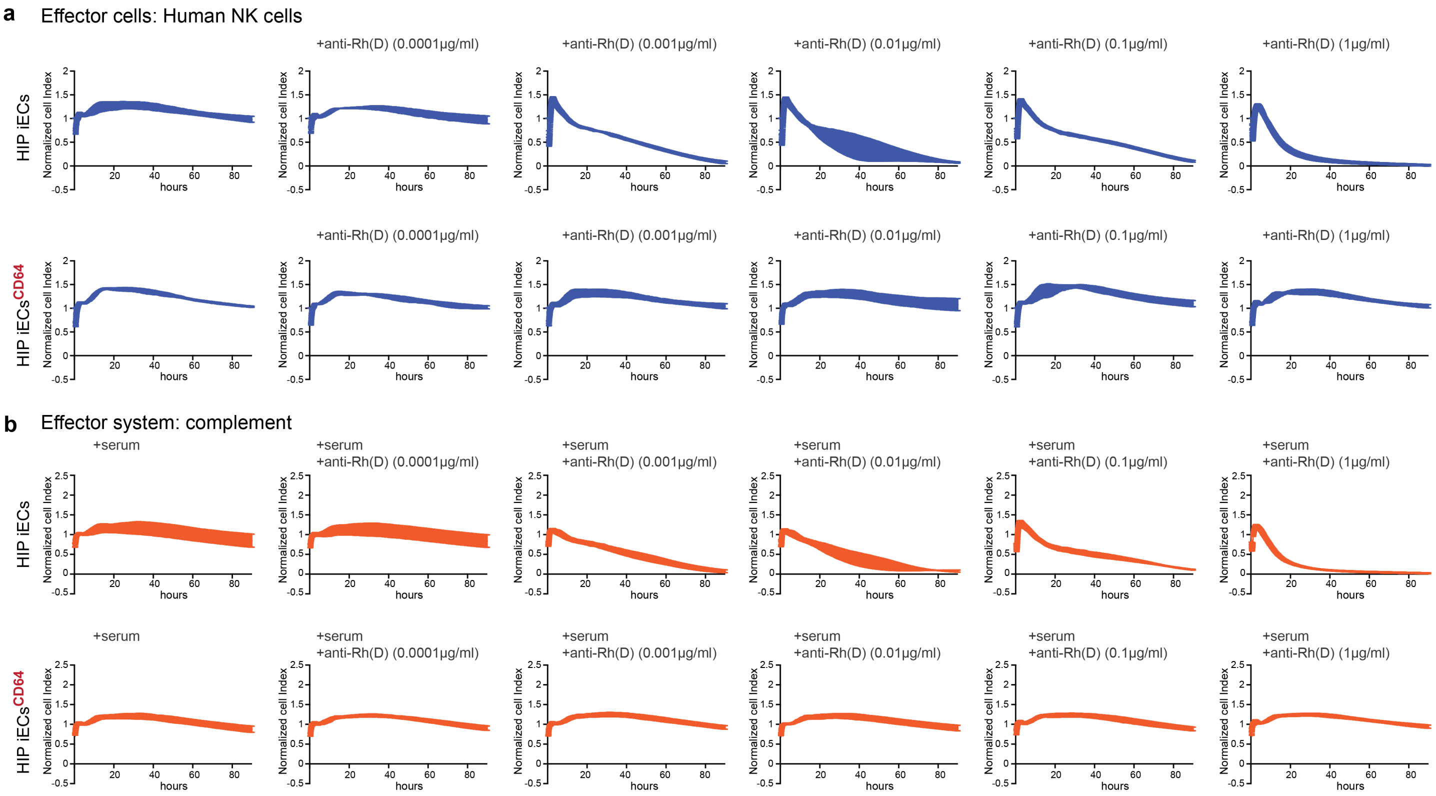


**Supplementary Figure 4**: *CD64 expression protects human HIP iECs from rhesus blood type antibody killing.* **a-b**, Human HIP iECs and HIP iECs$^{CD64}$ were challenged in impedance NK cell ADCC (a) and CDC (b) assays with different concentrations of an anti-Rh(D) IgG1 antibody (mean ± SD, three independent replicates per group and time point).

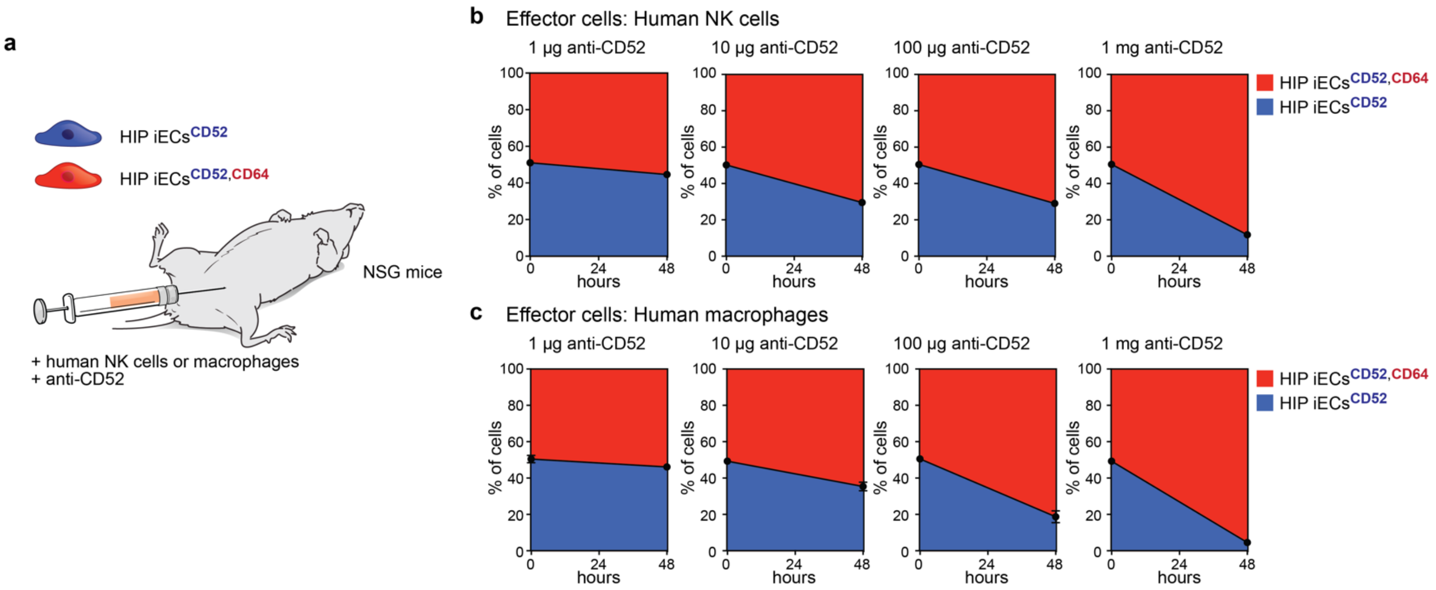


**Supplementary Figure 5**: *CD64 expression protects human HIP iECs$^{CD52,CD64}$ from non-HLA antibody NK cell and macrophage killing in vivo*. **a**, A 1:1 mixture of HIP iECs$^{CD52}$ and HIP iECs$^{CD52,CD64}$ was injected intraperitoneally into NSG mice together with allogeneic human NK cells or macrophages and alemtuzumab and was recovered 48 h later. **b-c**, The mixture was injected together with human NK cells (b) or macrophages (c) and the ratio of recovered cell populations is shown (mean ± SD, three independent replicates per group and time point).

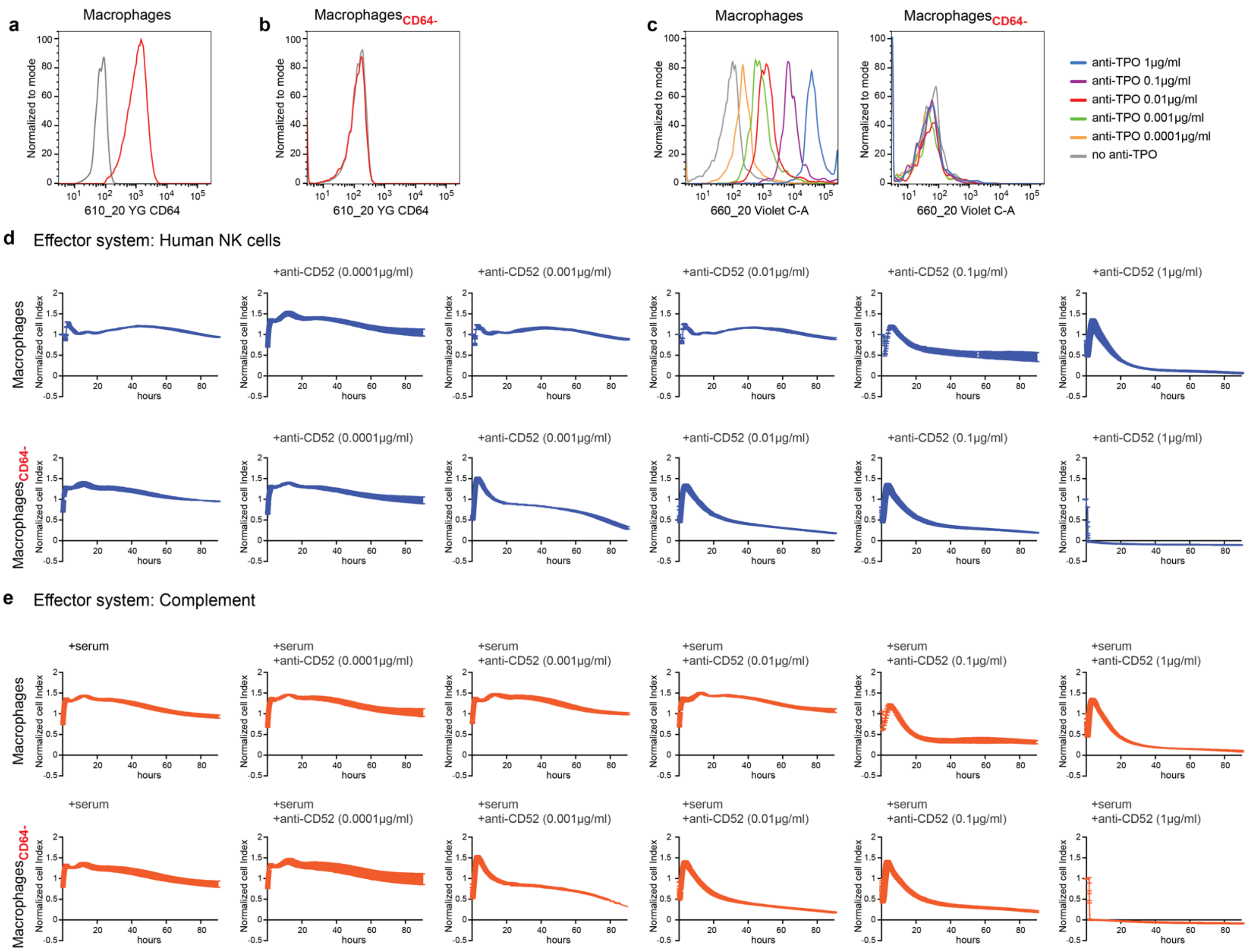


**Supplementary Figure 6**: *CD64-knockdown macrophages have increased susceptibility for ADCC and CDC killing.* **a-b**, Flow cytometry histograms for CD64 expression on human macrophages (a) and CD64-knockdown macrophages (b, macrophages$_{CD64-}$) (representative graphs of two independent experiments). **c**, Flow cytometry histograms for the binding of free IgG1 $F_c$ (anti-TPO) on macrophages and macrophages$_{CD64-}$ (representative graph of two independent experiments). **d-e**, Macrophages and macrophages$_{CD64-}$ were challenged in impedance NK cell ADCC (d) and CDC (e) assays with different concentrations of the anti-CD52 IgG1 antibody alemtuzumab (mean ± SD, three independent replicates per group and time point).

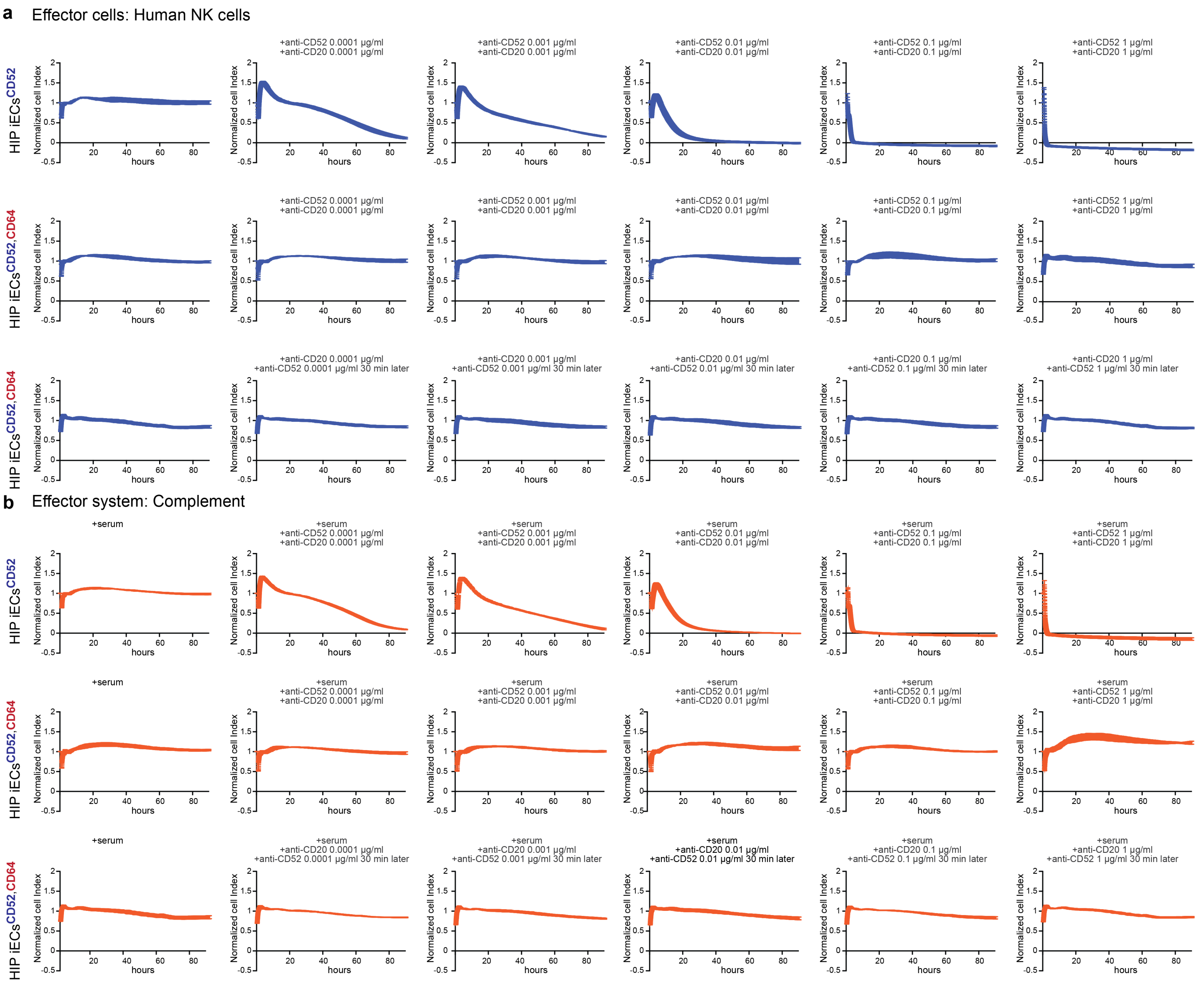


**Supplementary Figure 7**: *The $F_c$ capture mechanism works despite the presence of competing antibodies and serum.* **a-b**, Human HIP iECs$^{CD52}$ and HIP iECs$^{CD52,CD64}$ were challenged in impedance NK cell ADCC (a) and CDC (b) assays with different concentrations of two competing IgG1antibodies (anti-CD52 and anti-CD20). In some experiments, anti-CD52 was added 30 min after anti-CD20 (mean ± SD, three independent replicates per group and time point).

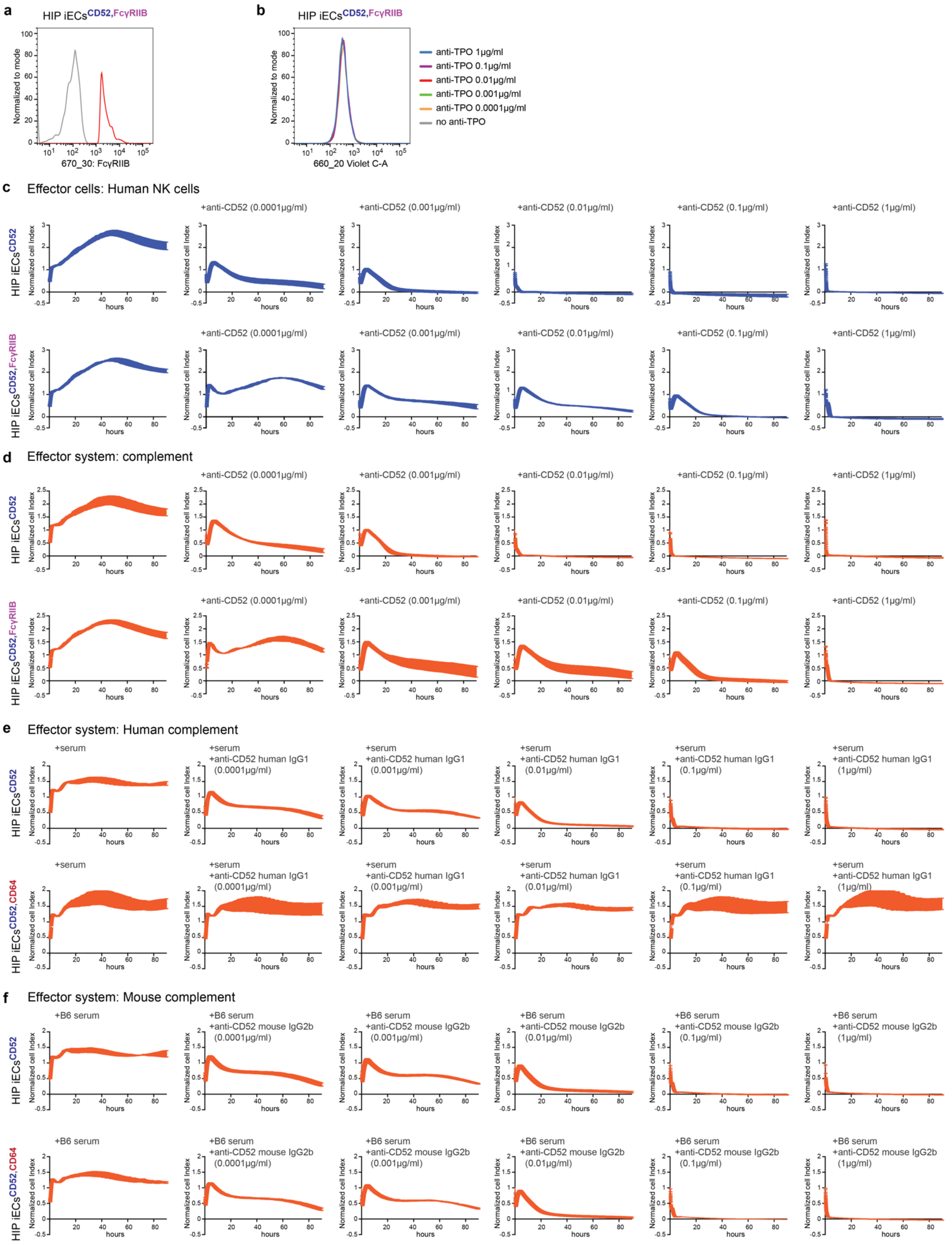


**Supplementary Figure 8**: *High-affinity IgG $F_c$ receptor binding is necessary to establish antibody protection.* **a**,

Flow cytometry histogram for FcγRIIB expression on human HIP iECs$^{CD52,Fc\gamma RIIB}$ (representative graphs of two independent experiments). **b**, Flow cytometry histograms for the binding of free IgG1 $F_c$ (anti-TPO) on human HIP iECs$^{CD52,Fc\gamma RIIB}$ (representative graph of two independent experiments). **c-d**, Human HIP iECs$^{CD52}$ and HIP iECs$^{CD52,Fc\gamma RIIB}$ were challenged in impedance NK cell ADCC (c) and CDC (d) assays with the anti-CD52 IgG1antibody alemtuzumab (mean ± SD, three independent replicates per group and time point). **e-f**, Human HIP iECs$^{CD52}$ and HIP iECs$^{CD52,CD64}$ were challenged in impedance NK cell ADCC and CDC assays with complement-fixing human anti-CD52 IgG1 (e) or a mouse anti-human CD52 IgG2b (f) in different concentrations (mean ± SD, three independent replicates per group and time point). The human IgG1 was used with human serum (e), the mouse IgG2b with mouse serum (f).

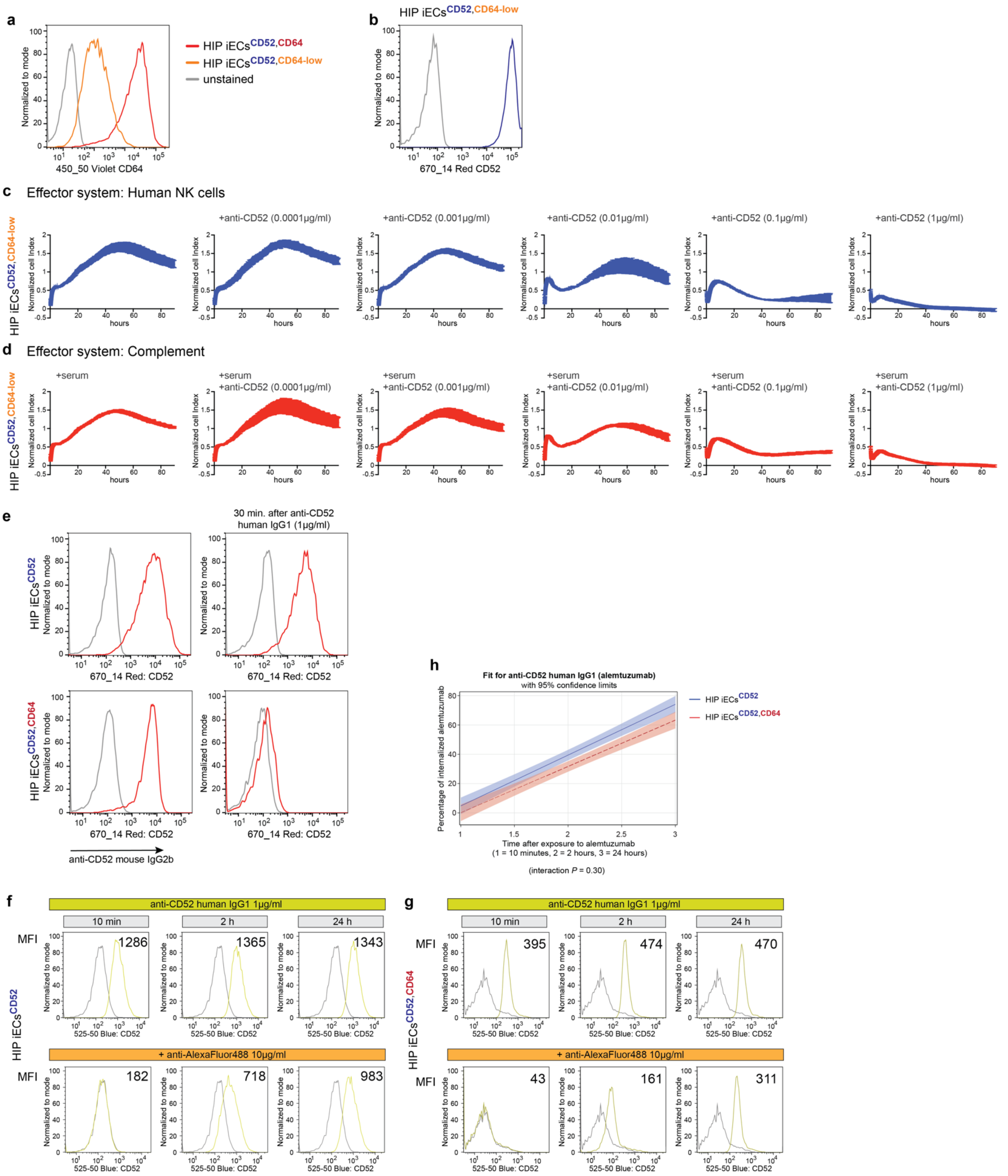


**Supplementary Figure 9**: *Antibody protection depends on expression levels of CD64 and involves occupation of target antigens.* **a,** Flow cytometry histograms for CD64 expression on HIP iEC$^{CD52,CD64}$ and HIP iEC$^{CD52,CD64-low}$ (representative graphs of two independent experiments). **b**, Flow cytometry histograms for CD52 expression

on HIP $iEC^{CD52,CD64\text{-}low}$ (representative graphs of two independent experiments). **c-d**, HIP $iEC^{CD52,CD64\text{-}low}$ were challenged in impedance NK cell ADCC (c) and CDC (d) assays with different concentrations of the anti-CD52 IgG1 antibody alemtuzumab (mean ± SD, three independent replicates per group and time point). With increasing antibody concentrations, there was increasingly rapid target cell killing. **e,** Flow cytometry histograms for the binding of 1 μg/ml anti-CD52 mouse IgG2b to HIP $iEC^{CD52}$ and HIP $iEC^{CD52,CD64}$ (representative graphs of two independent experiments) with and without prior incubation with 1 μg/ml human anti-CD52 IgG1 (alemtuzumab). Only on HIP $iEC^{CD52,CD64}$, pre-incubation with alemtuzumab mitigated the subsequent binding of the mouse IgG2b, which does not bind CD64 via $F_c$. **f-h**, The internalization of CD52-bound alemtuzumab over time was assessed in HIP $iEC^{CD52}$ (f) and HIP $iEC^{CD52,CD64}$ (g). AlexaFluor488-conjucated anti-CD52 IgG1 was incubated for different time periods before the fluorochrome was quenched using an anti-AlexaFluor488 antibody. The remaining fluorescence is specific for the internalized fluorochrome fraction. Linear regression analysis showed that the percentage of internalized alemtuzumab over time did not differ significantly by cell line (interaction $P = 0.30$, h).

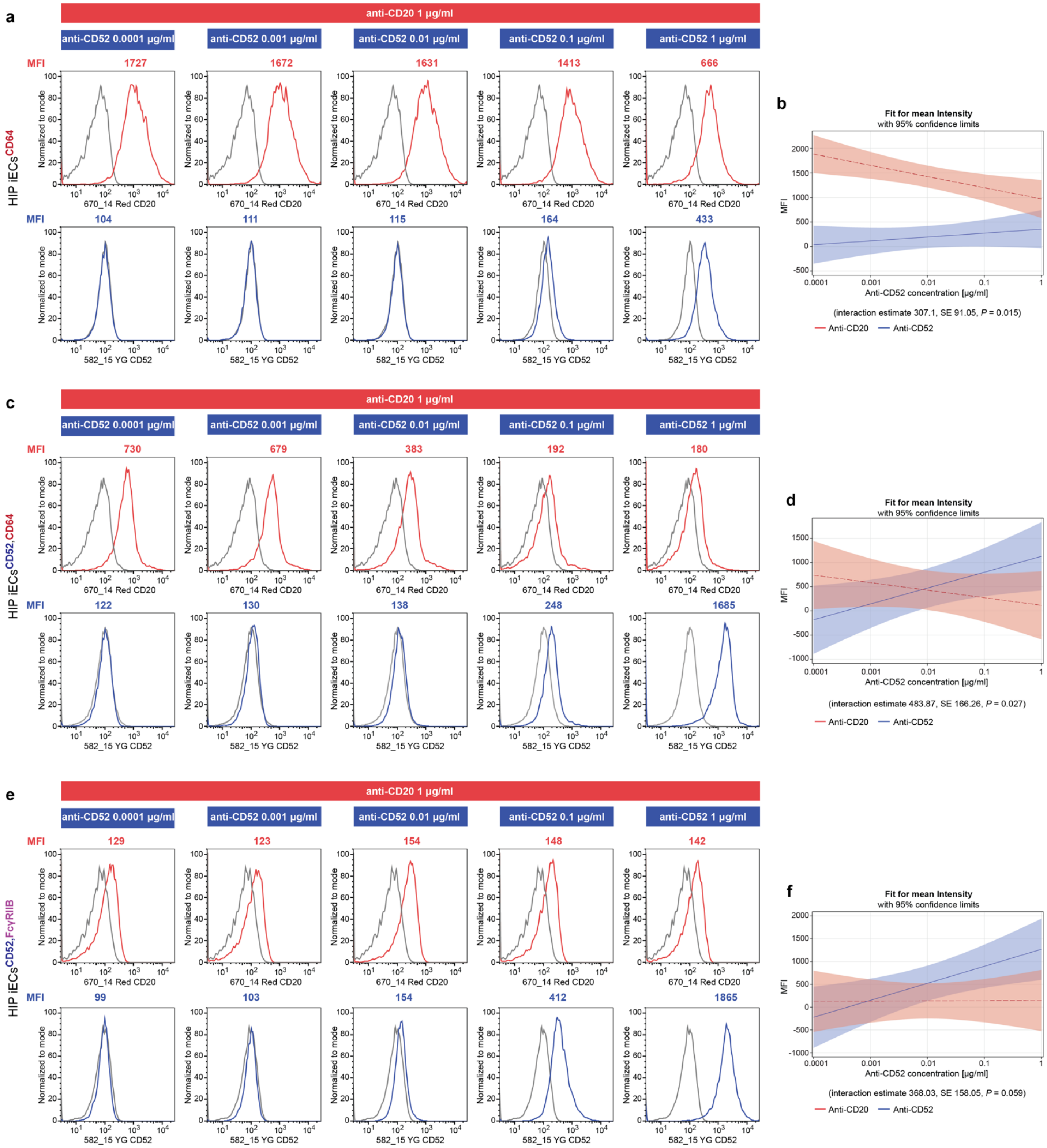


**Supplementary Figure 10**: *Competitive anti-CD20 vs. anti-CD52 binding.* Competitive binding of anti-CD52 and CD20 to HIP iECs$^{CD64}$ (a-b), HIP iECs$^{CD52,CD64}$ (c-d), and HIP iECs$^{CD52,Fc\gamma RIIB}$ (e-f) was determined by flow cytometry. Anti-CD20 was consistently used at 1 µg/ml while a range of anti-CD52 concentrations (0.0001 µg/ml to 1 µg/ml) was used (one representative histogram is shown). Linear regression plots for antibody interaction are shown (mean with 95% confidence limits, b, d, f).

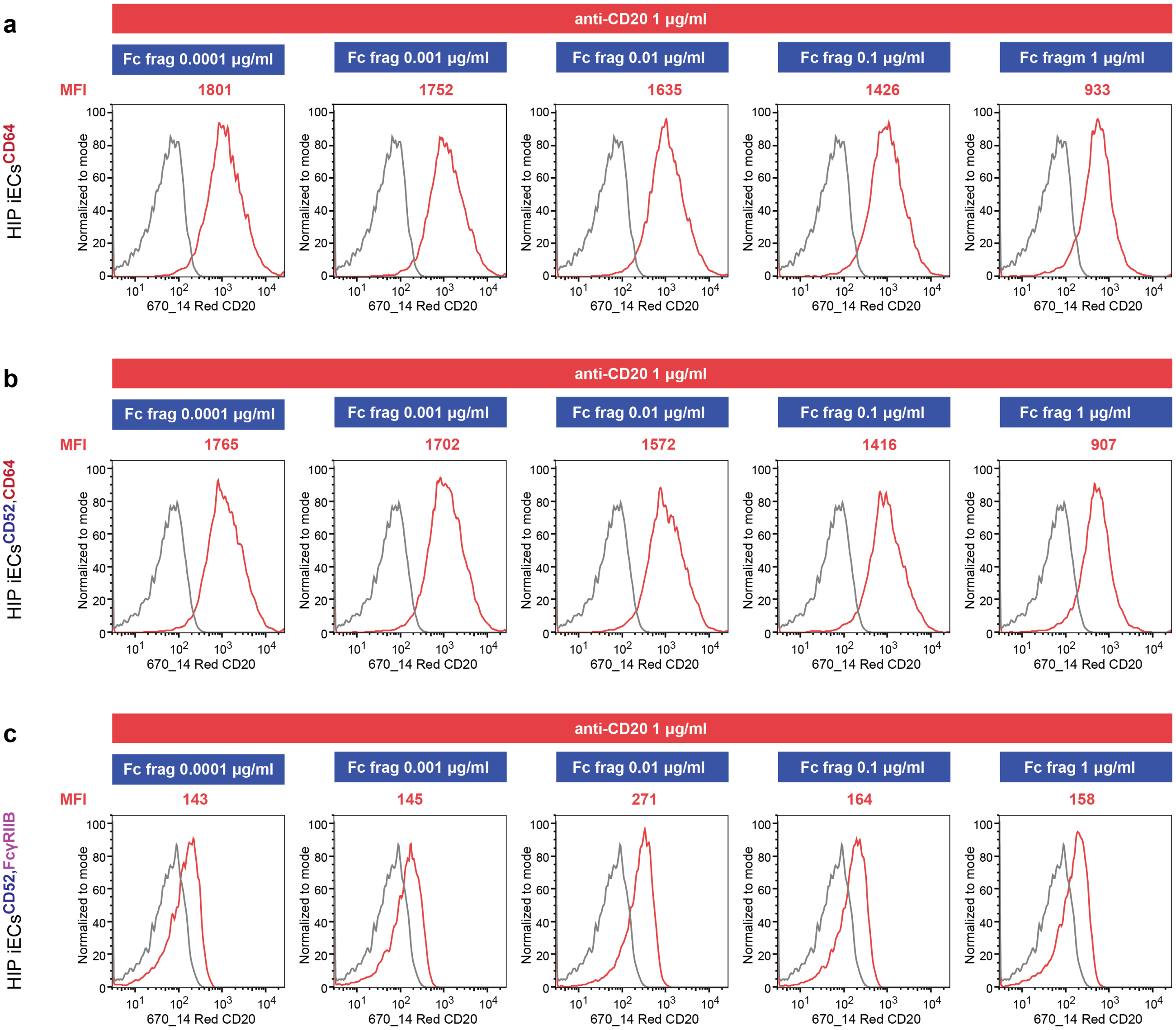


**Supplementary Figure 11**: *Competitive anti-CD20 vs. human IgG1 $F_c$ fragment.* The binding of 1 µg/ml anti-CD20 to HIP iECs$^{CD64}$ (a), HIP iECs$^{CD52,CD64}$ (b), and HIP iECs$^{CD52,Fc\gamma RIIB}$ (c) in the presence of human IgG1 $F_c$ fragment (0.0001 µg/ml to 1 µg/ml) was assessed by flow cytometry (one representative histogram of two independent experiments).

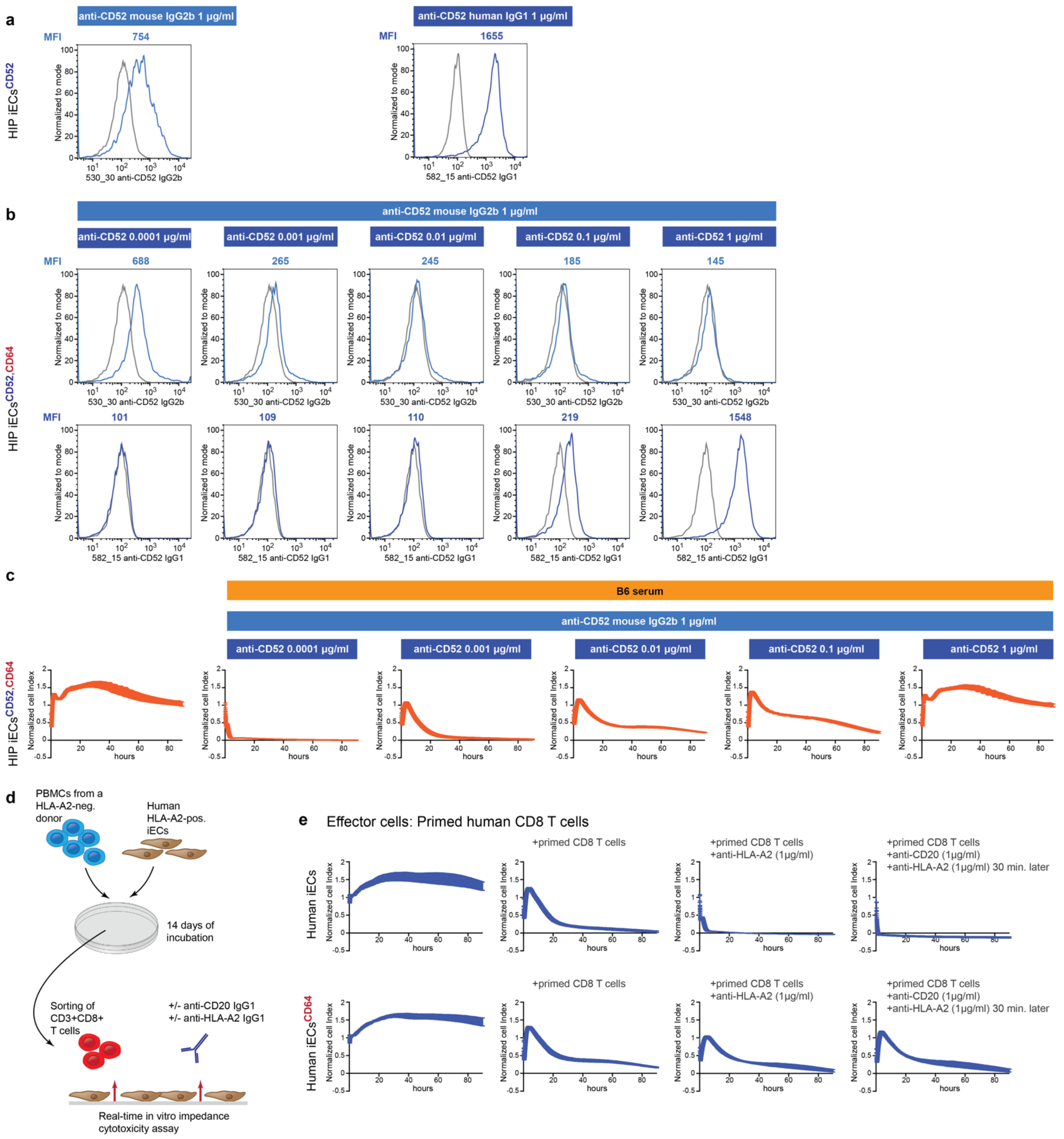


**Supplementary Figure 12**: *CD64 protects from CDC with competitive antibodies and from CD8⁺ T cell ADCC.*

**a-b**, Flow cytometry histograms showing the binding of anti-CD52 mouse IgG2b (a) and anti-CD52 human IgG1 (b) to HIP iECs$^{CD52}$ (representative graph of two independent experiments). **b**, Competitive binding of anti-CD52 mouse IgG2b and anti-CD52 human IgG1 to HIP iECs$^{CD52,CD64}$ was determined by flow cytometry. Anti-CD52 mouse IgG2b was consistently used at 1 µg/ml while anti-CD52 human IgG1 was used in a range of

concentrations (0.0001 μg/ml to 1 μg/ml, one representative histogram is shown). **c**, HIP iECs$^{CD52,CD64}$ were challenged in impedance CDC assays with complement-fixing anti-CD52 mouse IgG2b and human IgG1 in different concentrations (mean ± SD, three independent replicates per group and time point). Mouse serum was used in the CDC assays). **d**, PBMCs from an HLA-A2-negative donor were incubated with HLA-A2-positive iECs for 14 days to prime T cells. $CD8^+$ T cells were sorted by flow cytometry and used for subsequent killing assays. **e**, *In vitro* impedance $CD8^+$ T cell killing assays of human iECs and iECs$^{CD64}$ were performed in the absence and presence of 1 μg/ml anti-HLA-A2 IgG1, with or without previous incubation with a competing anti-CD20 IgG1 antibody (mean ± SD, three independent replicates per group and time point).

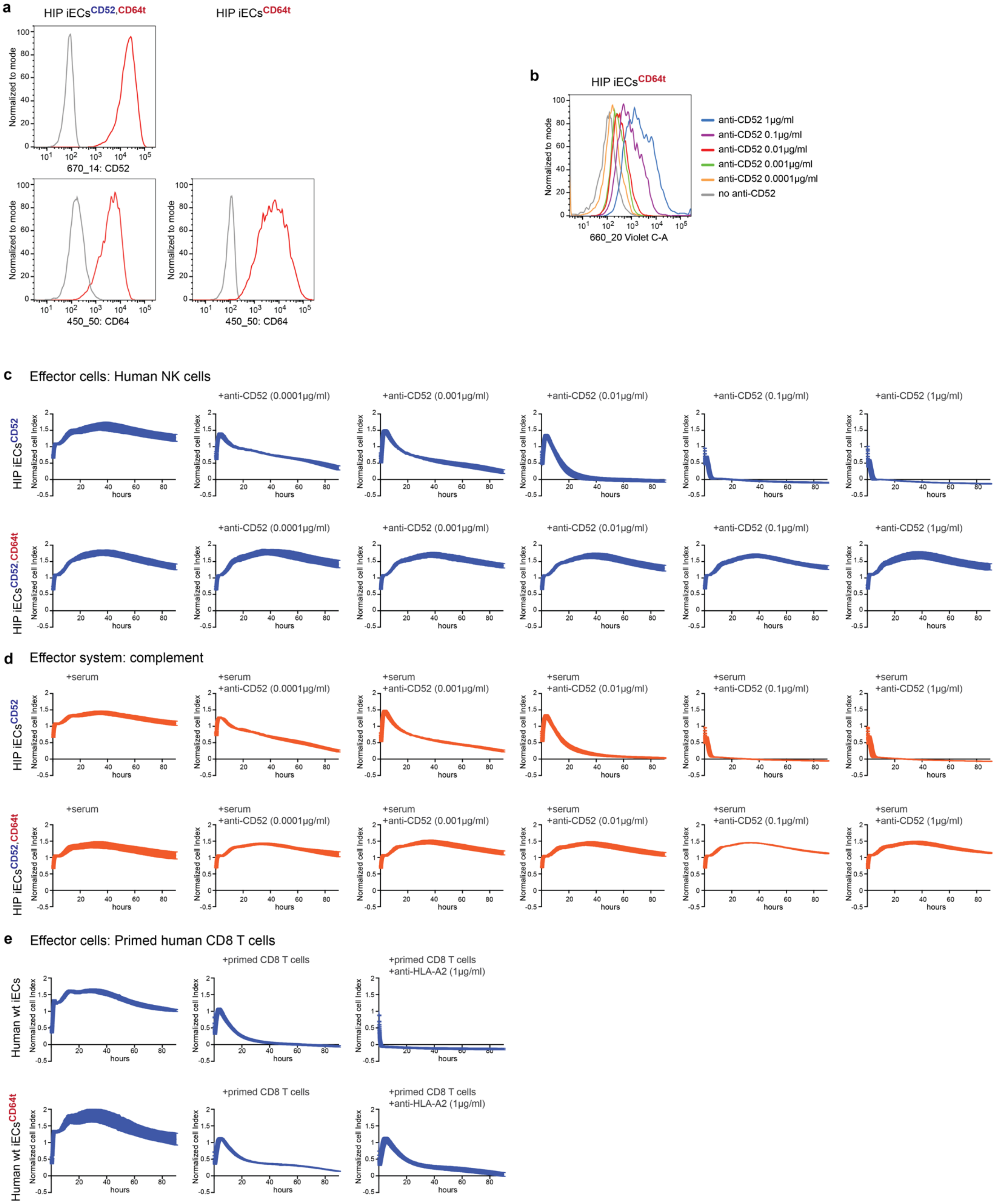


**Supplementary Figure 13**: *Truncated CD64 similarly protects from antibody-mediated killing*. **a**, Flow

cytometry histograms for CD52 and CD64t expression on HIP iEC$^{CD52}$ and HIP iEC$^{CD52,CD64t}$ (representative graphs of two independent experiments). **b**, Flow cytometry histograms for the binding of free IgG1 $F_c$ (anti-CD52, representative graph of two independent experiments). **c-d**, HIP iEC$^{CD52}$ and HIP iEC$^{CD52,CD64t}$ were challenged in impedance NK cell ADCC (c) and CDC (d) assays with different concentrations of the anti-CD52 IgG1 antibody alemtuzumab (mean ± SD, three independent replicates per group and time point). **e**, PBMCs from an HLA-A2-negative donor were incubated with HLA-A2-positive iECs for 14 days to prime T cells. CD8$^+$ T cells were sorted by flow cytometry and used for the killing assays. *In vitro* impedance CD8$^+$ T cell killing assays of human iECs and iECs$^{CD64t}$ were performed in the absence and presence of 1 μg/ml anti-HLA-A2 IgG1 (mean ± SD, three independent replicates per group and time point).

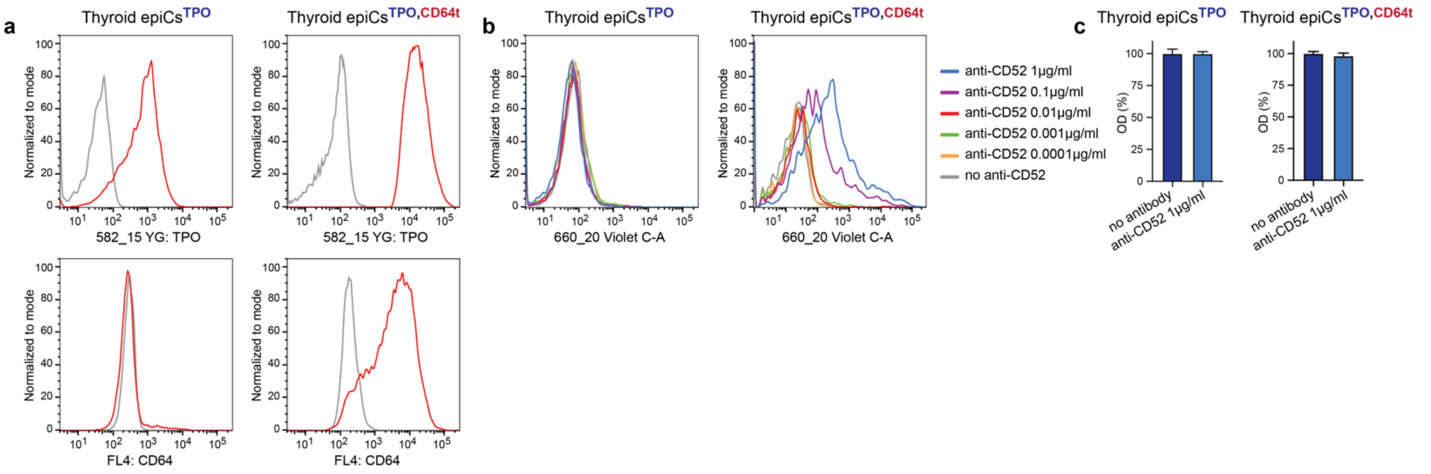


**Supplementary Figure 14**: *Characterization of human thyroid epiCs$^{TPO,CD64t}$*. **a**, Flow cytometry histograms for TPO and CD64t expression on human thyroid epiCs$^{TPO}$ and epiCs$^{TPO,CD64t}$ (representative graphs of two independent experiments). **b**, Flow cytometry histograms for the binding of free IgG1 $F_c$ (alemtuzumab, representative graph of two independent experiments). **c**, Thyroxine production by epiCs$^{TPO}$ and epiCs$^{TPO,CD64t}$ was measured in the presence or absence of 1 µg/ml anti-CD52 IgG1 antibodies using an ELISA (mean ± SD, three independent replicates per group and time point).

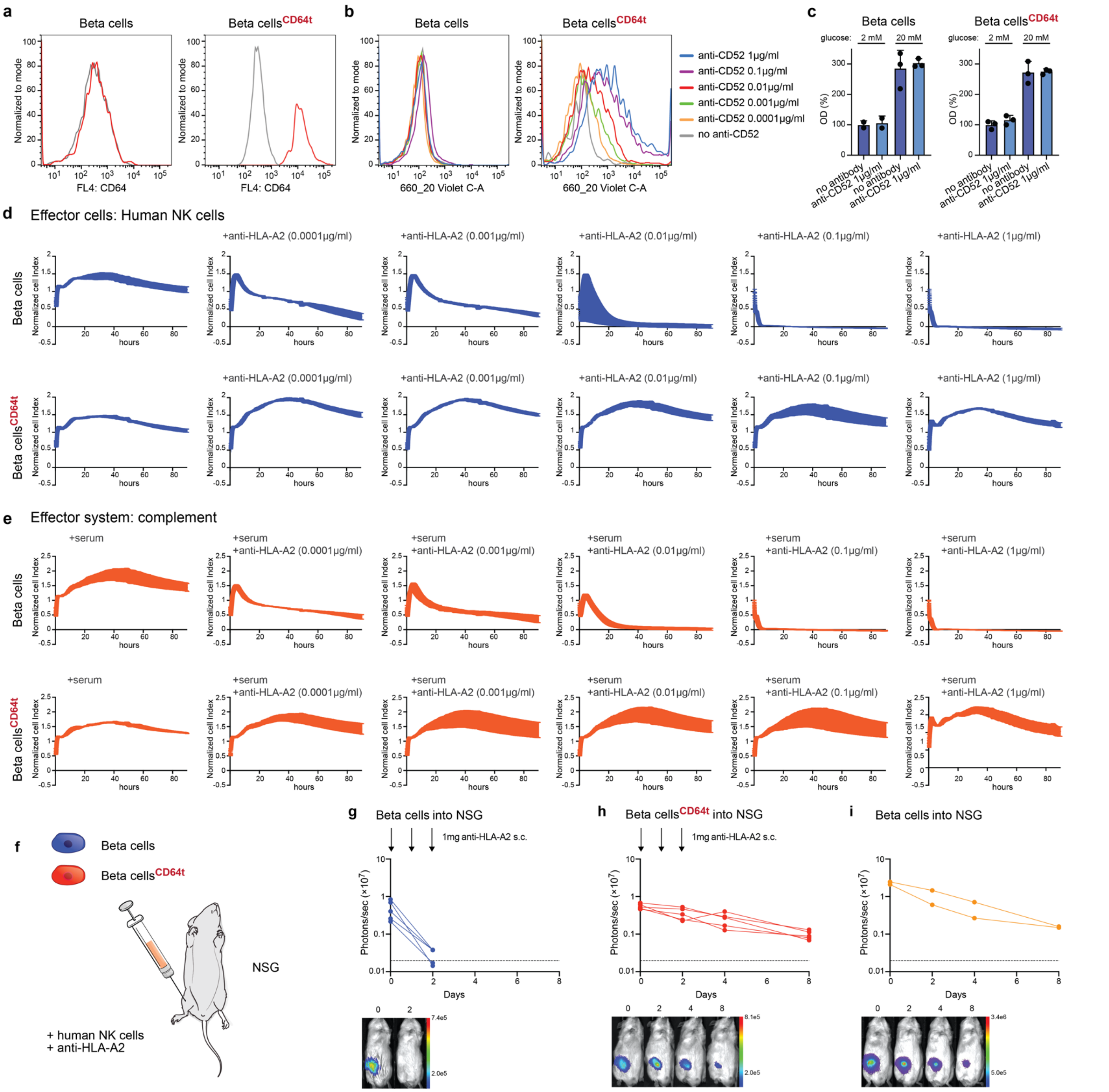


**Supplementary Figure 15**: *Human beta cells$^{CD64t}$ are protected from antibody-mediated killing*. **a**, Flow cytometry histogram for CD64t expression on human beta cells$^{CD64t}$ (representative graph of two independent experiments). **b**, Flow cytometry histogram for the binding of free IgG1 $F_c$ (alemtuzumab, representative graph of two independent experiments). **c**, Glucose sensing and insulin production by human beta cells and beta cells$^{CD64t}$ was measured in the presence or absence of 1 µg/ml anti-CD52 IgG1 antibodies using an ELISA (mean ± SD, two or three independent replicates per group and time point). The assays were performed under

low (2 mM) and high (20 mM) glucose conditions. **d-e**, Human beta cells and beta cells$^{CD64t}$ were challenged in impedance NK cell ADCC (d) and CDC (e) assays with different concentrations of an anti-HLA-A2 IgG1 antibody (mean ± SD, three independent replicates per group and time point). **f**, $5 \times 10^4$ human beta cells or beta cells$^{CD64t}$ were injected subcutaneously into NSG mice with $10^6$ human NK cells. Both groups received 3 subcutaneous 1 mg doses of anti-HLA-A2 IgG1 on days 0, 1, and 2. **g-h**, BLI signals of beta cells (g) and beta cells$^{CD64t}$ (h) were followed (all individual mice were plotted and BLI pictures of one representative mouse per group are shown). **i**, $5 \times 10^4$ human beta cells were injected subcutaneously into NSG mice without NK cells and without antibodies (all individual mice were plotted and BLI pictures of one representative mouse are shown).
