# Supplementary material for: Protection of cell therapeutics from antibody-mediated killing by CD64 overexpression
Source: Nat Biotechnol. 2023 Jan 2;41(5):717–27. doi: 10.1038/s41587-022-01540-7 (PMC10188358; doi:10.1038/s41587-022-01540-7)
Supplement: Supplementary file 2 — Reporting Summary [file 41587_2022_1540_MOESM2_ESM.pdf]

# nature portfolio

Corresponding author(s): Tobias Deuse

Last updated by author(s): Sep 26, 2022

# Reporting Summary

Nature Portfolio wishes to improve the reproducibility of the work that we publish. This form provides structure for consistency and transparency in reporting. For further information on Nature Portfolio policies, see our Editorial Policies and the Editorial Policy Checklist.

## Statistics

For all statistical analyses, confirm that the following items are present in the figure legend, table legend, main text, or Methods section.

| n/a | Confirmed | |
|---|---|---|
| ☐ | ☒ | The exact sample size (*n*) for each experimental group/condition, given as a discrete number and unit of measurement |
| ☐ | ☒ | A statement on whether measurements were taken from distinct samples or whether the same sample was measured repeatedly |
| ☐ | ☒ | The statistical test(s) used AND whether they are one- or two-sided<br>*Only common tests should be described solely by name; describe more complex techniques in the Methods section.* |
| ☒ | ☐ | A description of all covariates tested |
| ☒ | ☐ | A description of any assumptions or corrections, such as tests of normality and adjustment for multiple comparisons |
| ☐ | ☒ | A full description of the statistical parameters including central tendency (e.g. means) or other basic estimates (e.g. regression coefficient) AND variation (e.g. standard deviation) or associated estimates of uncertainty (e.g. confidence intervals) |
| ☐ | ☒ | For null hypothesis testing, the test statistic (e.g. *F*, *t*, *r*) with confidence intervals, effect sizes, degrees of freedom and *P* value noted<br>*Give P values as exact values whenever suitable.* |
| ☒ | ☐ | For Bayesian analysis, information on the choice of priors and Markov chain Monte Carlo settings |
| ☒ | ☐ | For hierarchical and complex designs, identification of the appropriate level for tests and full reporting of outcomes |
| ☒ | ☐ | Estimates of effect sizes (e.g. Cohen's *d*, Pearson's *r*), indicating how they were calculated |

*Our web collection on statistics for biologists contains articles on many of the points above.*

## Software and code

Policy information about availability of computer code

| | |
|---|---|
| Data collection | FlowJo 10 was used to analyze flow cytometric data. Prism9 was used for graphing and statistical analysis. Aura 3.2 was used for quantification of bioluminescence imaging. XCelligence assays were measured with the RTCA software. |
| Data analysis | Data were automatically analyzed in the mentioned software above for RTCA. Statistical analysis was performed on Prism9 or SAS version 9.4. |

For manuscripts utilizing custom algorithms or software that are central to the research but not yet described in published literature, software must be made available to editors and reviewers. We strongly encourage code deposition in a community repository (e.g. GitHub). See the Nature Portfolio guidelines for submitting code & software for further information.

## Data

Policy information about availability of data

All manuscripts must include a data availability statement. This statement should provide the following information, where applicable:

- Accession codes, unique identifiers, or web links for publicly available datasets
- A description of any restrictions on data availability
- For clinical datasets or third party data, please ensure that the statement adheres to our policy

All data generated or analyzed during this study are included in this published article (and its supplementary information files). No pre-established data exclusion method was used. No clinical data were included. Supplementary Information is available in the online version of the paper.

# Field-specific reporting

Please select the one below that is the best fit for your research. If you are not sure, read the appropriate sections before making your selection.

☒ Life sciences ☐ Behavioural & social sciences ☐ Ecological, evolutionary & environmental sciences

For a reference copy of the document with all sections, see nature.com/documents/nr-reporting-summary-flat.pdf

# Life sciences study design

All studies must disclose on these points even when the disclosure is negative.

| | |
|---|---|
| Sample size | The sample size for the in vivo studies to achieve statistical significance was not calculated before the studies as the survival of the HIP cells with or without human CD64 or mouse Cd64 in the different models was unknown prior. It was reasoned that 5-6 mice per group in individual experiments would indicate valid efficacy. No statistical test was used for the in vivo studies. Sample sizes in vitro were determined by three or more samples for comparisons between one or multiple groups, followed by the statistical test, where indicated. Again, the sample size to achieve statistical significance was not calculated before the studies for the reason described above. |
| Data exclusions | No pre-established data exclusion method was used. |
| Replication | The experimental findings can be reliably reproduced. Some key data generated by one co-author were repeated by other co-authors. The figure legends specify how often the experiments had been repeated. |
| Randomization | Some samples were number coded until the readout was finalized. The numbers were assigned prior to the experiment and determined the group/ treatment/ condition. Mice were number coded and randomly assigned to a group prior to the surgical procedure. |
| Blinding | For in vivo imaging, investigators doing the readouts were blinded and referred to the mice by their assigned numbers, which could later reveal the group they were in. For in vitro studies, usually different investigators performed the assay or did the analyses. The statistician is part of the UCSF core facility, not part of our lab, and unfamiliar with the science. |

# Reporting for specific materials, systems and methods

We require information from authors about some types of materials, experimental systems and methods used in many studies. Here, indicate whether each material, system or method listed is relevant to your study. If you are not sure if a list item applies to your research, read the appropriate section before selecting a response.

## Materials & experimental systems

| n/a | Involved in the study | |
|---|---|---|
| ☐ | ☒ | Antibodies |
| ☐ | ☒ | Eukaryotic cell lines |
| ☒ | ☐ | Palaeontology and archaeology |
| ☐ | ☒ | Animals and other organisms |
| ☒ | ☐ | Human research participants |
| ☒ | ☐ | Clinical data |
| ☒ | ☐ | Dual use research of concern |

## Methods

| n/a | Involved in the study | |
|---|---|---|
| ☒ | ☐ | ChIP-seq |
| ☐ | ☒ | Flow cytometry |
| ☒ | ☐ | MRI-based neuroimaging |

## Antibodies

| | |
|---|---|
| Antibodies used | Flow cytometry antibodies for mouse cells: BV421-labeled anti-mouse Cd64 (FcγRI) antibody (clone X54-5/7.1, Biolegend, catalog no. 139309), BV421-labeled anti-human CD64 antibody (clone 10.1, BD Biosciences, catalog no. 305002), APC-conjugated anti-human CD52 antibody (clone HI186, Biolegend, catalog no. 316008), anti-MHC class I (clone AF6-88.5.5.3, eBioscience, catalog no. 46-5958-82) or mouse IgG2a isotype-matched control antibody (clone eBM2a, eBioscience, catalog no. 46-4724-80), anti-MHC class II (clone M5/114.15.2, eBioscience, catalog no. 46-5321-82) or mouse IgG2b isotype-matched control antibody (clone eB149/10H5, eBioscience, catalog no. 46-4031-80), anti-mouse-Cd47 (clone miap301, BD Biosciences, catalog no. 563584) or rat IgG2a isotype-matched control antibody (clone R35-95, BD Biosciences, catalog no. 557690).<br><br>Flow cytometry antibodies for human cells: APC-conjugated anti-human CD52 antibody (clone HI186, Biolegend, catalog no. 316008), BV421-labeled anti-human CD64 antibody (clone 10.1, BD Biosciences, catalog no. 562872), PE-conjugated anti-human TPO antibody (clone MoAb47, Santa Cruz Biotechnology, catalog no. sc-58432), FITC-conjugated anti-FMC63 scFv (clone Y45, Acro Biosystems, catalog no. FM3-FY45), APC-conjugated anti-HLA-A,B,C antibody (clone G46_2.6,BD Biosciences, catalog no. 562006) or APC-conjugated IgG1 isotype-matched control antibody (clone MOPC-21, BD Biosciences, catalog no. 555751), Alexa-flour647-labeled anti-HLA-DR,DP,DQ antibody (clone Tu39, BD Biosciences, catalog no. 563591) or Alexa-flour647-labeled IgG2a isotype-matched control antibody (clone G155-178, BD Biosciences, catalog no. 557715), PerCP-Cy5.5-conjugated anti-CD47 (clone B6H12, BD Biosciences, catalog no. 561261) or PerCP-Cy5.5-conjugated IgG1 isotype-matched control antibody (clone MOPC-21, BD Biosciences, catalog no. 552834), humanized anti-MICA IgG1 (Creative Biolabs, catalog no. TAB-0799CL) with PE-labeled mouse anti-human IgG1 |

Fc secondary antibody (clone HP6001, Southern Biotech, catalog no. 9054-09), APC-conjugated anti-human CD32B/C antibody (clone S18005H, Biolegend, catalog no. 398303) AlexaFluor488-conjucated anti-CD52 (clone Hu116, catalog no. FAB9889G, R&D Systems), anti-AlexaFluor488 quenching antibody (Invitrogen, catalog no. A-11094). To study competing CD52 binding, an APC-conjugated anti-CD52 mouse IgG2b was used (clone HI186, catalog no. GTX80134, GeneTex, Irvine, CA).

Antibodies to assess mouse Cd64-Fc binding: mouse IgG2a anti-CD20 (clone rIGEL/773, Abcam, ab219329) and QDot655-labeled F(ab')2-goat anti-mouse IgG secondary antibody (ThermoFisher, catalog no. Q-11021MP). Antibodies to assess human CD64-Fc binding: humanized IgG1 anti-CD52 (alemtuzumab, ichorbio, catalog no. ICH4002) and QDot655-labeled F(ab')2-goat anti-human IgG secondary antibody (Thermo Fisher Scientific, catalog no. Q-11221MP).

Antibodies to assess human CD64-Fc binding: humanized IgG1 anti-CD52 (alemtuzumab, ichorbio, catalog no. ICH4002), humanized IgG1 anti-TPO (clone B8, Creative Biolabs, catalog no. FAMAB-0014JF) and QDot655-labeled F(ab')2-goat anti-human IgG secondary antibody (Thermo Fisher Scientific, catalog no. Q-11221MP), anti-SIRPalpha (clone KWAR23) human IgG1, IgG2, IgG3, and IgG4 (Creative Biolabs, custom order).

NK cell MACS sorting: The MagniSort Mouse NK cell Enrichment Kit (Invitrogen) was used followed by CD49b MACS-sorting (Miltenyi).

Antibodies for mouse in vitro killing assays: mouse IgG2a anti-H-2b (BioXCell, clone AF6-88.5.5.3, catalog no. BE0121), humanized anti-CD52 IgG1 (alemtuzumab, ichorbio, catalog no. ICH4002).

Antibodies for human in vitro killing assays: humanized anti-CD52 IgG1 (alemtuzumab, ichorbio, catalog no. ICH4002), humanized anti-MICA IgG1 (Creative Biolabs, catalog no. TAB-0799CL), humanized anti-HLA-A2 IgG1 (clone 3PF12, Absolute Antibody, catalog no. AB00947-10.0), humanized anti-Rh(D) IgG1 (clone F5, Creative Biolabs, catalog no. FAMAB-0089WJ), humanized anti-TPO IgG1 (clone B8, Creative Biolabs, catalog no. FAMAB-0014JF), humanized anti-CD3 IgG1 (Creative Biolabs, custom product), humanized anti-CD19 scFv (FMC63) IgG1 (clone 136.20.1, Creative Biolabs, catalog no. HPAB-0440-YJ-m/h), mouse anti-human CD52 IgG2b (MyBioSource, catalog no. MBS4158863).

Antibodies for in vivo killing assays: humanized anti-CD52 IgG1 (alemtuzumab, ichorbio, catalog no. ICH4002), humanized anti-HLA-A2 IgG1 (clone 3PF12, Absolute Antibody, catalog no. AB00947-10.0), humanized anti-TPO IgG1 (clone B8, Creative Biolabs, catalog no. FAMAB-0014JF).

Validation

Each antibody was tested with positive and negative control prior to staining the samples. Isotype and tested antibody were concentration matched. Antibody concentration were gathered from vendors datasheets:
MACS sorting
Anti-CD15 MACS-sorting (Miltenyi, https://www.miltenyibiotec.com/US-en/products/cd15-microbeads-human.html#gref), CD49b MACS-sorting (Miltenyi, https://www.miltenyibiotec.com/US-en/products/cd49b-antibody-anti-mouse-reafinity-rea541.html#apc:30-ug-in-200-ul).

Antibodies
BV421-labeled anti-mouse Cd64 (FcγRI) antibody (clone X54-5/7.1, Biolegend, catalog no. 139309, https://www.biolegend.com/en-us/products/brilliant-violet-421-anti-mouse-cd64-fcgammari-antibody-8992), BV421-labeled anti-human CD64 antibody (clone 10.1, BD Biosciences, catalog no. 562872, https://www.bdbiosciences.com/en-ca/products/reagents/flow-cytometry-reagents/research-reagents/single-color-antibodies-ruo/bv421-mouse-anti-human-cd64.562872), APC-conjugated anti-human CD52 antibody (clone HI186, Biolegend, catalog no. 316008, https://www.biolegend.com/en-us/products/apc-anti-human-cd52-antibody-3947), anti-MHC class I (clone AF6-88.5.5.3, eBioscience, catalog no. 46-5958-82, https://www.thermofisher.com/antibody/product/MHC-Class-I-H-2Kb-Antibody-clone-AF6-88-5-5-3-Monoclonal/46-5958-82), mouse IgG2a isotype-matched control antibody (clone eBM2a, eBioscience, catalog no. 46-4724-80, https://www.thermofisher.com/antibody/product/Mouse-IgG2a-kappa-clone-eBM2a-Isotype-Control/46-4724-80), anti-MHC class II (clone M5/114.15.2, eBioscience, Santa Clara, CA, catalog no. 46-5321-82, https://www.thermofisher.com/antibody/product/MHC-Class-II-I-A-I-E-Antibody-clone-M5-114-15-2-Monoclonal/46-5321-82), rat IgG2b isotype-matched control antibody (clone eB149/10H5, eBioscience, catalog no. 46-4031-80, https://www.thermofisher.com/antibody/product/Rat-IgG2b-kappa-clone-eB149-10H5-Isotype-Control/46-4031-80), anti-Cd47 (clone miap301, BD Biosciences, catalog no. 563584, https://www.bdbiosciences.com/en-ca/products/reagents/flow-cytometry-reagents/research-reagents/single-color-antibodies-ruo/alexa-fluor-647-rat-anti-mouse-cd47.563584), rat IgG2a isotype-matched control antibody (clone R35-95, BD Biosciences, catalog no. 557690, https://www.bdbiosciences.com/en-ca/products/reagents/flow-cytometry-reagents/research-reagents/flow-cytometry-controls-and-lysates/alexa-fluor-647-rat-igg2a-isotype-control.557690), APC-conjugated anti-human CD52 antibody (clone HI186, Biolegend, catalog no. 316008, https://www.biolegend.com/en-us/products/apc-anti-human-cd52-antibody-3947), BV421-labeled anti-human CD64 antibody (clone 10.1, BD Biosciences, catalog no. 562872, https://www.bdbiosciences.com/en-ca/products/reagents/flow-cytometry-reagents/research-reagents/single-color-antibodies-ruo/bv421-mouse-anti-human-cd64.562872), PE-conjugated anti-human TPO antibody (clone MoAb47, Santa Cruz Biotechnology, catalog no. sc-58432, https://www.scbt.com/p/thyroperoxidase-antibody-moab47), or FITC-conjugated anti-FMC63 scFv (clone Y45, Acro Biosystems, catalog no. FM3-FY45, https://www.acrobiosystems.com/P3228-FITC-Labeled-Monoclonal-Anti-FMC63-scFv-Antibody-Mouse-IgG1-%28Y45%29-DMF-Filed.html), BV421 mouse anti-human CD64 antibody (clone 10.1, catalog no. 562872, BD Biosciences, https://www.bdbiosciences.com/en-ca/products/reagents/flow-cytometry-reagents/research-reagents/single-color-antibodies-ruo/bv421-mouse-anti-human-cd64.562872), isotype-matched control mouse IgG1 antibody (clone MOPC-21, catalog no. 400157, Biolegend, https://www.biolegend.com/en-us/products/brilliant-violet-421-mouse-igg1-kappa-isotype-ctrl-7194), APC mouse anti-human CD32B/C antibody (clone S18005H, catalog no. 398304, Biolegend, https://www.biolegend.com/en-us/products/apc-anti-human-cd32bc-antibody-19460), isotype-matched control APC mouse IgG1 antibody (clone MOPC-21, catalog no. 555751, BD Biosciences, https://www.bdbiosciences.com/en-ca/products/reagents/flow-cytometry-reagents/research-reagents/flow-cytometry-controls-and-lysates/apc-mouse-igg1-isotype-control.555751), APC-conjugated anti-HLA-A,B,C antibody (clone G46_2.6,BD Biosciences, catalog no. 562006, https://www.bdbiosciences.com/en-ca/products/reagents/flow-cytometry-reagents/research-reagents/single-color-antibodies-ruo/apc-mouse-anti-human-hla-abc.562006), APC-conjugated IgG1 isotype-matched control antibody (clone MOPC-21, BD Biosciences, catalog no. 555751, https://www.bdbiosciences.com/en-ca/products/reagents/flow-cytometry-reagents/research-reagents/flow-cytometry-controls-and-lysates/apc-mouse-igg1-isotype-control.555751). Alexa-flour647-labeled anti-HLA-DR,DP,DQ antibody (clone Tu39, BD Biosciences, catalog no. 563591, https://www.bdbiosciences.com/en-ca/products/reagents/flow-cytometry-reagents/research-reagents/single-color-antibodies-ruo/alexa-fluor-647-mouse-anti-human-hla-dr-dp-dq.563591), Alexa-flour647-labeled IgG2a isotype-matched control antibody (clone G155-178, BD Biosciences, catalog no.

557715, https://www.bdbiosciences.com/en-ca/products/reagents/flow-cytometry-reagents/research-reagents/flow-cytometry-controls-and-lysates/alexa-fluor-647-mouse-igg2a-isotype-control.557715), PerCP-Cy5.5-conjugated anti-CD47 (clone B6H12, BD Biosciences, catalog no. 561261, https://www.bdbiosciences.com/en-ca/products/reagents/flow-cytometry-reagents/research-reagents/single-color-antibodies-ruo/percp-cy-5-5-mouse-anti-human-cd47.561261), PerCP-Cy5.5-conjugated IgG1 isotype-matched control antibody (clone MOPC-21, BD Biosciences, catalog no. 552834, https://www.bdbiosciences.com/en-ca/products/reagents/flow-cytometry-reagents/research-reagents/flow-cytometry-controls-and-lysates/percp-cy-5-5-mouse-igg1-isotype-control.552834), anti-MICA IgG1 (Creative Biolabs, catalog no. TAB-0799CL, https://www.creativebiolabs.net/Anti-MICA-Recombinant-Antibody-TAB-0799CL-27487.htm), PE-labeled mouse anti-human IgG1 Fc secondary antibody (clone HP6001, Southern Biotech, catalog no. 9054-09, https://www.southernbiotech.com/mouse-anti-human-igg1-fc-pe-hp6001-9054-09), APC-labeled mouse anti-human SIRP[illegible] (clone 15-414, catalog no. 372106, Biolegend, https://www.biolegend.com/en-us/products/apc-anti-human-cd172a-sirpalpha-antibody-14165), IgG2a, [illegible] isotype-matched control antibody (catalog no. 557715, BD Biosciences, https://www.bdbiosciences.com/en-ca/products/reagents/flow-cytometry-reagents/research-reagents/flow-cytometry-controls-and-lysates/alexa-fluor-647-mouse-igg2a-isotype-control.557715), APC mouse anti-human CD3 antibody (clone SP34-2, catalog no. 557597, BD Biosciences, https://www.bdbiosciences.com/en-ca/products/reagents/flow-cytometry-reagents/research-reagents/single-color-antibodies-ruo/apc-mouse-anti-human-cd3.557597), isotype-matched control APC mouse IgG1[illegible] antibody (clone MOPC-21, catalog no. 550854, BD Biosciences, https://www.bdbiosciences.com/en-ca/products/reagents/flow-cytometry-reagents/research-reagents/flow-cytometry-controls-and-lysates/apc-mouse-igg1-isotype-control.550854), BV421 mouse anti-human CD8 antibody (clone SK1, catalog no. 344748, Biolegend, https://www.biolegend.com/en-us/products/brilliant-violet-421-anti-human-cd8-antibody-13512), isotype-matched control mouse IgG1[illegible] antibody (clone MOPC-21, catalog no. 400157, Biolegend, https://www.biolegend.com/en-us/products/brilliant-violet-421-mouse-igg1-kappa-isotype-ctrl-7194), QDot655-labeled F(ab')2-goat anti-mouse IgG secondary antibody (ThermoFisher, catalog no. Q-11021MP, https://www.thermofisher.com/antibody/product/Goat-anti-Mouse-IgG-H-L-Secondary-Antibody-Polyclonal/Q-11021MP), QDot655-labeled F(ab')2-goat anti-human IgG secondary antibody (Thermo Fisher Scientific, catalog no. Q-11221MP, https://www.thermofisher.com/antibody/product/Goat-anti-Human-IgG-H-L-Secondary-Antibody-Polyclonal/Q-11221MP), QDot655-labeled F(ab')2-goat anti-human IgG secondary antibody (Thermo Fisher Scientific, catalog no. Q-11221MP, https://www.thermofisher.com/antibody/product/Goat-anti-Human-IgG-H-L-Secondary-Antibody-Polyclonal/Q-11221MP), mouse IgG2a anti-CD20 (clone rIGEL/773, Abcam, ab219329, https://www.abcam.com/cd20-antibody-rigel773-ab219329.html), humanized IgG1 anti-CD52 (alemtuzumab, ichorbio, catalog no. ICH4002, https://ichor.bio/product/alemtuzumab-biosimilar-research-grade-ich4002/), mouse IgG2a anti-H-2b (BioXCell, clone AF6-88.5.5.3, catalog no. BE0121, https://bxcell.com/product/h-2-k-b-2/), anti-FMC63 scFv (clone Y45, Acro Biosystems, catalog no. FM3-FY45, https://www.acrobiosystems.com/P3228-FITC-Labeled-Monoclonal-Anti-FMC63-scFv-Antibody-Mouse-IgG1-%28Y45%29-DMF-Filed.html), APC-conjugated anti-CD52 mouse IgG2b was used (clone HI186, catalog no. GTX80134, GeneTex, Irvine, CA, https://www.genetex.com/Product/Detail/CD52-antibody-HI186-APC/GTX80134), humanized IgG1 anti-TPO (clone B8, Creative Biolabs, catalog no. FAMAB-0014JF, https://www.creativebiolabs.net/anti-tpo-fab-fragment-clone-b8-137834.htm), AlexaFluor488-conjugated anti-CD52 (clone Hu116, catalog no. FAB9889G R&D Systems, https://www.rndsystems.com/products/human-cd52-research-grade-alemtuzumab-biosimilar-alexa-fluor-488-conjugated-antibody-hu116_fab9889g), anti-AlexaFluor488 quenching antibody (Invitrogen, catalog no. A-11094, https://www.thermofisher.com/antibody/product/Alexa-Fluor-488-Antibody-Polyclonal/A-11094), humanized anti-MICA IgG1 (Creative Biolabs, catalog no. TAB-0799CL, https://www.creativebiolabs.net/Anti-MICA-Recombinant-Antibody-TAB-0799CL-27487.htm), humanized anti-HLA-A2 IgG1 (clone 3PF12, Absolute Antibody, catalog no. AB00947-10.0, https://absoluteantibody.com/product/anti-hla-a2a28-3pf12/), humanized anti-Rh(D) IgG1 (clone F5, Creative Biolabs, catalog no. FAMAB-0089WJ, https://www.creativebiolabs.net/anti-rh-d-fab-fragment-clone-f5-131552.htm), humanized anti-TPO IgG1 (clone B8, Creative Biolabs, catalog no. FAMAB-0014JF, https://www.creativebiolabs.net/anti-tpo-fab-fragment-clone-b8-137834.htm), humanized anti-CD3 IgG1 (Creative Biolabs, custom product), humanized anti-CD19 scFv (FMC63) IgG1 (clone 136.20.1, Creative Biolabs, catalog no. HPAB-0440-YJ-m/h, https://www.creativebiolabs.net/anti-cd19-scfv-fmc63-recombinant-antibody-hpab-0440-yj-122546.htm).

# Eukaryotic cell lines

Policy information about cell lines

**Cell line source(s)**

The Human Episomal iPSC Line was purchased from Thermo Fisher Scientific (Waltham, MA). Mouse iPSCs were reprogramed from C57BL/6 mice. Irradiated CF1 Mouse Embryonic Fibroblasts (MEFs) were used as feeder cells for mouse iPSCs and purchased from Thermo Fisher Scientific. Immortalized human thyroid epithelial cells (epiCs) were purchased from InScreenEx (catalog no. INS-CI-1017, Germany). Human iPSC-derived pancreatic beta cells were purchased from TaKaRa (ChiPSC22, catalog no. Y10106).

**Authentication**

None of the cell lines used have been authenticated.

**Mycoplasma contamination**

All cell lines were tested and negative for mycoplasma contamination using the Universal Mycoplasma test kit from ATCC.

**Commonly misidentified lines**
(See ICLAC register)

No commonly misidentified cell lines were used.

# Animals and other organisms

Policy information about studies involving animals; ARRIVE guidelines recommended for reporting animal research

**Laboratory animals**

C57BL/6 (C57BL/6J, B6, H2b, 000664, male, 6-12 weeks old), Rag-1 KO (B6.129S7-Rag1tm1Mom/J, 002216, male, 6-12 weeks old), and NSG (NOD.Cg-Prkdcscid Il2rgtm1Wjl/SzJ, 005557, female, 12-20 weeks old) and humanized NSG-SGM3 mice (NOD.Cg-Prkdcscid Il2rgtm1Wjl Tg(CMV-IL3,CSF2,KITLG)1Eav/MloySzJ, 013062, female, 12-20 weeks old) were purchased from the Jackson Laboratories

| | |
|---|---|
| | (Sacramento, CA). Mice were housed in 12 hour light-dark cycles with humidity between 30-70% at ambient temperature of 68-79 degrees Fahrenheit. |
| Wild animals | No wild animals were used |
| Field-collected samples | No field collected samples were used |
| Ethics oversight | Mice received humane care in compliance with the Guide for the Principles of Laboratory Animals. Animal experiments were approved by the University of California San Francisco (UCSF) Institutional Animal Care and Use Committee and performed according to local guidelines. |

Note that full information on the approval of the study protocol must also be provided in the manuscript.

# Flow Cytometry

## Plots

Confirm that:

- [x] The axis labels state the marker and fluorochrome used (e.g. CD4-FITC).
- [x] The axis scales are clearly visible. Include numbers along axes only for bottom left plot of group (a 'group' is an analysis of identical markers).
- [x] All plots are contour plots with outliers or pseudocolor plots.
- [x] A numerical value for number of cells or percentage (with statistics) is provided.

## Methodology

| | |
|---|---|
| Sample preparation | Cells were counted, stained and measured as single cell suspension in PBS+2% FCS hi. |
| Instrument | Cells were analyzed on the LSRFortessa (BD Bioscience) and results were expressed as fold-change to isotype-matched control Ig staining. |
| Software | The FlowJo software was used. |
| Cell population abundance | For flow cytometry analysis, more than 10,000 positive cells were measured. Cell sorting was gated for the desired population and sorted for the cell amount needed for assays. |
| Gating strategy | Samples were gated in FSC/SSC for the correct cell size and live cells. Isotype was measured for each sample as defined as unspecific staining threshold. |

- [ ] Tick this box to confirm that a figure exemplifying the gating strategy is provided in the Supplementary Information.
